# Chloroplast genome structure analysis of *Equisetum* unveils phylogenetic relationships to ferns and mutational hotspot region

**DOI:** 10.3389/fpls.2024.1328080

**Published:** 2024-04-11

**Authors:** Weiyue Sun, Zuoying Wei, Yuefeng Gu, Ting Wang, Baodong Liu, Yuehong Yan

**Affiliations:** ^1^ Key Laboratory of Plant Biology, College of Heilongjiang Province, Harbin Normal University, Harbin, China; ^2^ Key Laboratory of National Forestry and Grassland Administration for Orehid Conservation and Utilization, the Orchid Conservation & Research Center of Shenzhen, Shenzhen, China; ^3^ Key Laboratory of Plant Resources Conservation and Sustainable Utilization, South China Botanical Garden, Guangzhou, China

**Keywords:** phylogenomics, divergent hotspot, evolutionary, sequence characteristic, pteridophytes

## Abstract

*Equisetum* is one of the oldest extant group vascular plants and is considered to be the key to understanding vascular plant evolution. *Equisetum* is distributed almost all over the world and has a high degree of adaptability to different environments. Despite the fossil record of horsetails (*Equisetum*, Equisetaceae) dating back to the Carboniferous, the phylogenetic relationship of this genus is not well, and the chloroplast evolution in *Equisetum* remains poorly understood. In order to fill this gap, we sequenced, assembled, and annotated the chloroplast genomes of 12 species of *Equisetum*, and compared them to 13 previously published vascular plants chloroplast genomes to deeply examine the plastome evolutionary dynamics of *Equisetum*. The chloroplast genomes have a highly conserved quadripartite structure across the genus, but these chloroplast genomes have a lower GC content than other ferns. The size of *Equisetum* plastomes ranges from 130,773 bp to 133,684 bp and they encode 130 genes. Contraction/expansion of IR regions and the number of simple sequences repeat regions underlie large genomic variations in size among them. Comparative analysis revealed we also identified 13 divergence hotspot regions. Additionally, the genes *accD* and *ycf1* can be used as potential DNA barcodes for the identification and phylogeny of the genus *Equisetum*. Twelve photosynthesis-related genes were specifically selected in *Equisetum*. Comparative genomic analyses implied divergent evolutionary patterns between *Equisetum* and other ferns. Phylogenomic analyses and molecular dating revealed a relatively distant phylogenetic relationship between *Equisetum* and other ferns, supporting the division of pteridophyte into Lycophytes, Equisetaceae and ferns. The results show that the chloroplast genome can be used to solve phylogenetic problems within or between *Equisetum* species, and also provide genomic resources for the study of *Equisetum* systematics and evolution.

## 1 Introduction

Horsetails are considered an enigmatic group among terrestrial plants. While they consist solely of the genus *Equisetum*, they are tracing back to the Triassic period believed to be one of the oldest surviving genus within vascular plants (Hauke, 1963, 1979; Husby et al., 2011). Scientists have long been intrigued by this genus, recognizing its significance in unraveling the evolution of vascular plants (Hauke, 1979). Although its morphology and anatomy have been well documented (Bierhorst, 1958; Stein, 1993), the position of the genus in the plant life tree has puzzled researchers since the beginning of the 20th century (Scott, 1900; Browne, 1908; Eames, 1936; Rothwell, 1999; Pryer et al., 2001; Karol et al., 2010; Rothfels et al., 2015). The phylogeny position of *Equisetum* has traditionally been determined using morphological characters, as well as various chloroplast segments and the nuclear ribosomal ITS markers (Des Marais et al., 2003; Guillon, 2007; Grewe et al., 2013; Christenhusz et al., 2019). However, previous studies have faced challenges in establishing conclusive relationships of *Equisetum*, particularly when relying on a limited number of samples. Chloroplasts genome play a key role in the adaptation and evolution of plants (Wicke et al., 2011; Yin et al., 2018; Gao et al., 2019; Thode and Lohmann, 2019). The chloroplast genomes are self-replicating and evolve slowly (Parks et al., 2012; Li et al., 2019). Chloroplast genomes which have conserved structures and stable gene compositions, are among the smallest genomes in plant cells (Parks et al., 2009; Ruhlman and Jansen, 2014; Sun et al., 2017; Yan et al., 2018). They also provide easily accessible full sequences. Consequently, they have been widely applied in reconstructing the Tree of Life of plants, especially concerning lower taxonomic levels and peripheral categories (Jansen et al., 2007; Moore et al., 2007; Ruhfel et al., 2014; Gitzendanner et al., 2018; Shen et al., 2018; Li et al., 2019). Particularly, comparative analysis based on chloroplast genome data can provide insight into species evolution and phylogeny (Ruhfel et al., 2014).

The study of comparative genomics using chloroplast genome has mainly focused on structural variations, such as IR contractions and expansions (Guisinger et al., 2011; Zhu et al., 2016; Weng et al., 2017) and genomic rearrangements (Knox, 2014; Sun et al., 2017; Rabah et al., 2019). In addition to discovering sequence variation, comparative chloroplast genome can also identifying mutational hotspot regions. For species identification and population genetics, mutational hotspot regions and SSRs can be effective molecular markers (Dong et al., 2012). Chloroplast genomes can also provide a window into the process of adaptation, as many chloroplast genes are under strong selection of one kind or another. An example demonstrating significant implications for the adaptation of the *Ostreobium quekettii* to exceedingly low light levels was the discovery of prominent purifying selection instead of the anticipated positive selection in its chloroplast genome (R Marcelino et al., 2016). However, there have been no reports on the comparison of chloroplast genomes in the study of vascular plant *Equisetum*. This research significantly contributed to enhancing our comprehension regarding the adaptive evolution of plants.

The main objective of this research was to gain a thorough understanding of the evolutionary patterns exhibited by the chloroplast genomes among various *Equisetum* species. We sequenced, spliced, assembled, and annotated the chloroplasts from a total of 12 *Equisetum* species. To further analysis, we compared these genomes with 13 available chloroplast genomes of other vascular plants found in the GenBank database. The aims of this research were to exhibit the initial chloroplast genome information for 7 *Equisetum* species, and utilize all accessible *Equisetum* chloroplast genome sequences to elucidate their phylogenetic relationships, compare the genome structure and diversity for chloroplast genomes, explore single-sequence repeats and hotspot regions sequences for *Equisetum* species recognition and phylogenetic, and detect genes that have undergone positive selection and potential indications of adaptive evolution in the genus.

## 2 Manuscript formatting

### 2.1 Plant materials and sequencing

The identification of the species investigated in this study was determined by consulting the Flora of China database. **Supplementary Table 1** contains the sample data, which includes eleven recently sequenced species and fourteen species obtained from the National Center for Biotechnology Information (NCBI). We gathered fresh leaves and then desiccated them using silica gel for future DNA isolation. Novogene (Beijing, China) utilized the NovaSeq 6000 platform with 2 × 150 bp sequencing to analyze the isolated DNA using short reads.

### 2.2 Genome annotation and assembly

To ensure the removal of low-quality sequences and junctions, Trimmomatic (v0.39) was employed for quality control on the obtained raw reads from sequencing (Bolger et al., 2014). We conducted the assembly of the filtered reads by employing GetOrganelle (v1.6.2) the kmers were set to 21, 45, 65, 85, and 105 (Jin et al., 2020). Next, we finalized the assembly process using Bandage (v0.8.1) (Wick et al., 2015). Published *E. arvense* (JN968380.1), *E. hyemale* (NC_020146.1) and *E. ramosissimum* (MW074919.1) chloroplast genome was used as references. The annotation of the chloroplast genome was performed using PGA (Qu et al., 2019) and then reviewed and edited manually in Geneious v11.0.4 (Kearse et al., 2012). Ultimately, using Chloroplot to visualize the circular genome maps of the *Equisetum* species (Lohse et al., 2013).

### 2.3 Repeats analysis and variation detection

We utilized the Repter software to identify scattered repetitive sequences, having a min length of 3 bp and allowing a max of 30 bp nucleotide mismatches (Zheng, 2020). Retrieve repeated sequences using four methods: forward, backward, completion, and palindrome. The size of gaps between repeat sequences is limited to a maximum length of 3 kb. Use online MISA software (https://webblast.ipk-gatersleben.de/misa) to detect SSR (Thiel et al., 2008). In the scientific analysis, MISA was utilized for the anticipation of SSRs utilizing the subsequent parameters: monomer (n ≥8), dimer (n ≥4), trimer (n ≥4), tetramer (n ≥3), pentamer (n ≥3), hexamer (n ≥3). An SSR was defined as a combination of SSRs that were spaced apart by a max distance of 100 bp. To determine the types and amounts of substitution and indel mutations in the LSC, SSC, and IR regions of *Equisetum*, the software Dnasp (v5.10) was utilized. Manual counting and identification are necessary for inversion mutations (Librado and Rozas, 2009).

### 2.4 Chloroplast genome alignment

To analyze the IR boundary characteristics in the chloroplast genomes of Equisetum species, the utilization of IRscope facilitated the creation of the IR boundary map (Amiryousefi et al., 2018). To identify rearrangements and inverse evolutionary events, we employed the Mauve Contig Mover (MCM) alignment algorithm in Geneious (Darling et al., 2004). Using the shuffle-Lagan model, we utilized the mVISTA program to perform a comparison of the chloroplast genome sequences of all species (Frazer et al., 2004), used the *E. xylochaetum* as reference. Afterwards, DnaSP v5.10.01 was employed to calculate the nucleotide diversity (*Pi*) (Rozas et al., 2017). For this analysis, a window length of 600 bp and a step size of 200 bp were utilized.

### 2.5 Codon usage pattern

To avoid bias in the sampling process, we examined every protein coding gene (CDS) within the chloroplast genome. We ensured that each gene was complete, and both the start and stop codons were correctly present. Codon usage calculations excluded any CDS less than 300 base pairs in length to maintain accuracy. In order to evaluate the consistency of codon usage among synonymous codon, the ENc value was utilized. The evaluation of gene codon usage was conducted using the CodonW (v1.4.4) software, which provided us with three crucial indices: the Neutrality plot, GC3s, and ENc (Peden, 2000; Rosenberg et al., 2003).

### 2.6 Evolutionary selection pressure analysis

This study sequenced and annotated the chloroplast whole genome sequences of 12 species of the *Equisetum*, combined with the chloroplast gene sequences of 6 species ferns published in NCBI for a total of 18 species, to explore the role of selection pressure and adaptive evolution between the *Equisetum* and ferns, as well as the subgenus *Hippochaete* and *Equisetum*. In order to detect the sites under selection in the protein-coding genes in *Equisetum* plastid genomes, the nonsynonymous (Ka) and synonymous (Ks) nucleotide substitution rates and their ratio (ω=Ka/Ks) were calculated using the Codeml program in the PAML4.7 package (options were set to seqtype = 1, model = 0, NSsites = 0,1,2,3,7,8 in the codeml. ctl file) (Yang and Nielsen, 2002; Yang et al., 2005). PAML analyses were conducted in the “user tree” mode. The maximum likelihood (ML) phylogenetic evolutionary tree was obtained based on the complete chloroplast genomes using RA x ML (Stamatakis, 2006). We employed site specific models to analyze the selection pressure on 78 common protein-coding genes shared by all of the genomes. This model allowed the ω ratio to vary among sites with a fixed ω ratio in all the evolutionary branches. Other parameters in the CODEML control file were left at default settings. Two likelihood ratio tests were performed to check for the presence of positively selected sites: M1 (neutral) vs. M2 (positive selection), and M7 (beta) vs.M8 (beta and ω), which were compared using site-specific models (Yang and Nielsen, 2002; Yang et al., 2005).

### 2.7 Phylogenetic analysis

A total of 28 chloroplast genomes were selected to construct phylogenetic trees, with species *Anthoceros punctatus, Marchantia polymorpha* and *Physcomitrella patens* selected as the outgroups.. The involved the extract chloroplast whole genome, Genes, CDS, and chloroplast genome Noncoding sequences separately and use the four data sets phylogenetic analyses and utilization of MAFFT software to identify homologous sequences within the chloroplast genome of the 28 selected species. IQtree was used to construct ML tree, and 1000 bootstrap tests were run under the best nucleic acid replacement model. After the ML tree is obtained, the branch results are evaluated by bootstrap (BS), BS ≥ 70%. The jModelTest software was used to select the substitution model of nucleic acid sequence after alignment, and the BIC was used. Bayesian information criterion is the standard to select the best model; Phylosuite software is used to generate the.nex format file needed to construct the BI tree. Then use MrBayes to build the BI tree, the parameters selected in the Mrbayes program: Generations selected 2,000,000 times, and the Sampling Freq was set to 100, that is, running 2,000,000 generations for testing, and collecting evolutionary trees every 100 generations. If the value of Average standard deviation of split frequencies is less than 0.01 after the set program is run, assuming the convergence of MCMC chain, the input command no is stopped according to the prompt, otherwise the input command yes continues to run until its ASDF value is less than 0.01, and the tree building command is MrBayes > execute example.nex. After the BI tree is obtained, Bayesian posterior probabilities (pp) are used to evaluate the branch results, and pp ≥ 0.95 indicates that the results are reliable. Due to the lack of high homology in chloroplast genome Noncoding sequences, we selected ASTRAL software was used to merge the gene trees constructed by Genes, CDSs and chloroplast genome sequences to reconstruct the corresponding species tree (Yin et al., 2019).

### 2.8 Divergence time estimates using fossil calibrations

In order to obtain the stem and crown age of the genus *Equisetum*, we performed divergence time estimation using the concatenated chloroplast whole genome, CDSs and Gene dataset in BEAST2 v2.6.3 (Bouckaert et al., 2019). The geological age of each fossil was utilized to assign age correction points through a lognormal distribution approach. The estimation of absolute divergence times was conducted by employing the BEAST software (v1.8.4) and implementing the Bayesian lognormal relaxation clock method (Rambaut et al., 2018). The GTR + F + I model for the combined data set was applied with a speciation tree and a relaxed clock log normal clock model after. In order to assess the impact of individual calibration points, we conducted multiple runs of BEAST, omitting a different fossil in each iteration. Three secondary correction points were set according to the results of Christenhusz et al. (2021): (a) the crown age of all *Equisetum* was 152-164 Ma (Channing et al., 2011), (b) The crown age of *Subgenus Hippochaete* is 66-72 Ma (Reed, 1971), (c) The crown age of *E variegatum* and *E ramosissimum* is 37.8–47.8 Ma (Brown, 1975). We ran Markov Chain Monte Carlo (MCMC) for 800 million generations with sampling every 1,000 cycles. The first 10% of samples were discarded as burn-in. We examined the outputs using Tracer v1.7 to confirm convergence based on ESS >200 (Rambaut et al., 2018).

## 3 Results

### 3.1 Chloroplast genome sequencing and characteristics analysis

**Figure 1:**
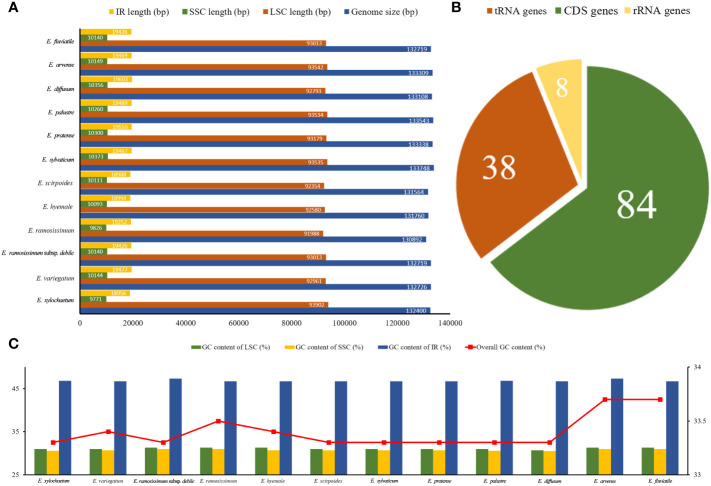
Summary data on the assembly of chloroplast genomes in *Equisetum*. **(A)** Size (bp). **(B)** Number of genes. **(C)** GC content (%).

**Figure 2:**
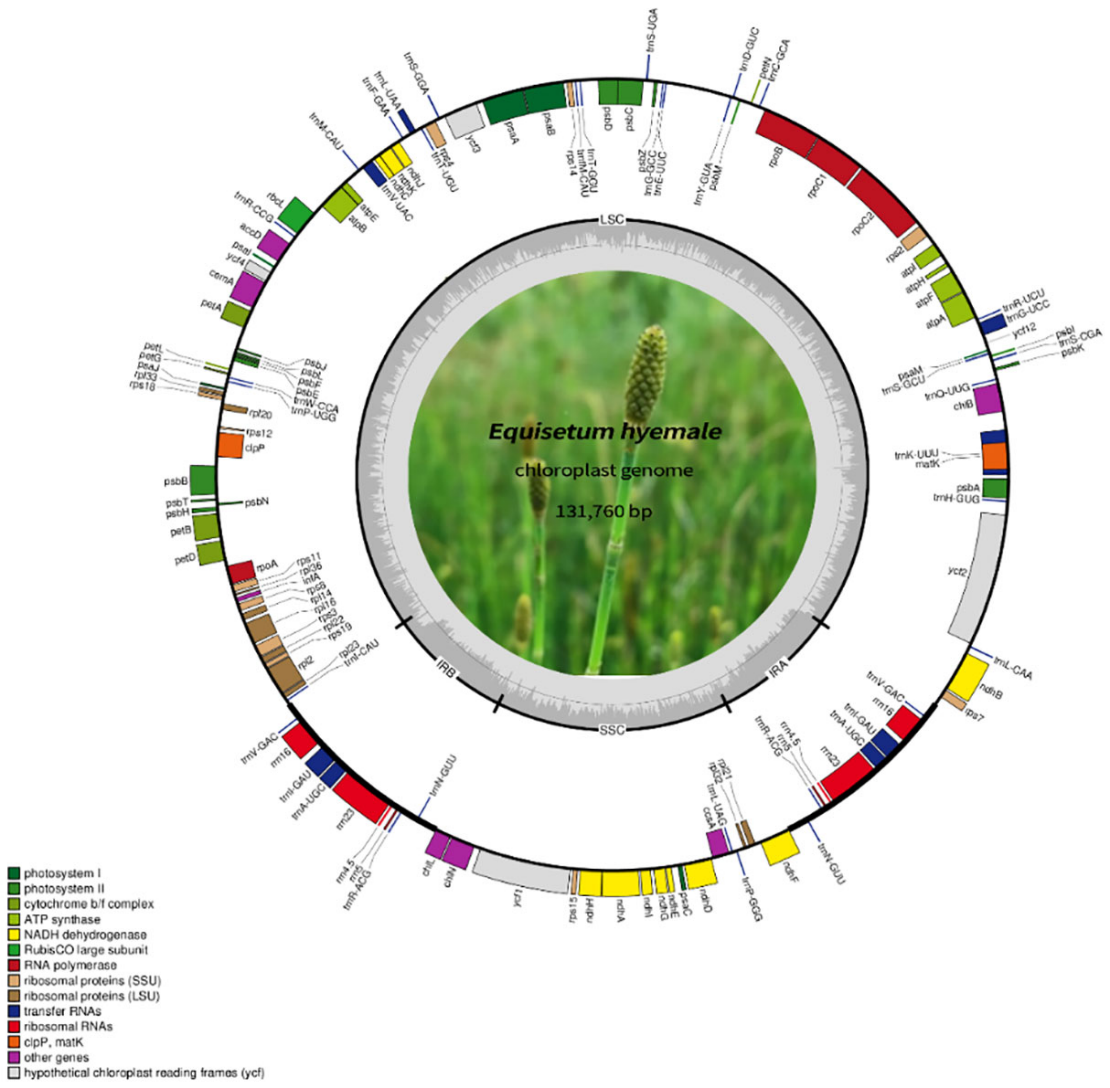
The comprehensive arrangement of the chloroplast genome in *Equisetum*. The large (LSC) and small (SSC) single copy regions are separated by the inverted repeats (IRa, IRb), represented by bold black lines on the inner circle. Genes located outside the circle undergo transcription in a counter-clockwise fashion, while those inside undergo transcription in a clockwise direction.

A total of 85.1 Gb clean data were obtained from the 12 *Equisetum* species, with genome sizes ranging from 130,773 bp (*E. hyemale*) to 133,684 bp (*E. arvense*) (**Figure 1A**). All of the 12 complete chloroplast genomes exhibited a characteristic structure consisting of four parts, which included a duo of IR regions (9,828 - 10,252 bp), LSC regions (91,524 - 93,536 bp), and SSC regions (18,941 - 19,855 bp). Compared with the outgroups, the *Equisetum* chloroplast genomes had lower GC content. The analysis of base composition suggests that the IR regions had higher GC content (46.7%) than LSC (33.1%) and SSC (30.7%) regions (**Figure 1C**). The gene annotations and raw data have already been submitted to NCBI Gen-Bank (**Figure 2**).

A sum of 130 annotated genes was identified within the chloroplast genomes of *Equisetum* species, encompassing 84 protein-coding genes, 8 rRNA genes, and 38 tRNA genes (**Figure 1B**). Gene expression regulation is significantly influenced by introns. According to **Supplementary Table 2**, there were duplicated copies of a combined total of 18 genes present in the *Equisetum* chloroplast genomes. This includes four rRNA genes (*rrn5, rrn4.5, rrn23, rrn16*) and five tRNA genes (*trnV-GAC, trnI-GAU, trnA-UGC, trnR-ACG, trnN-GUU*), each duplicated twice.

### 3.2 Phylogenetic relationships

**Figure 3:**
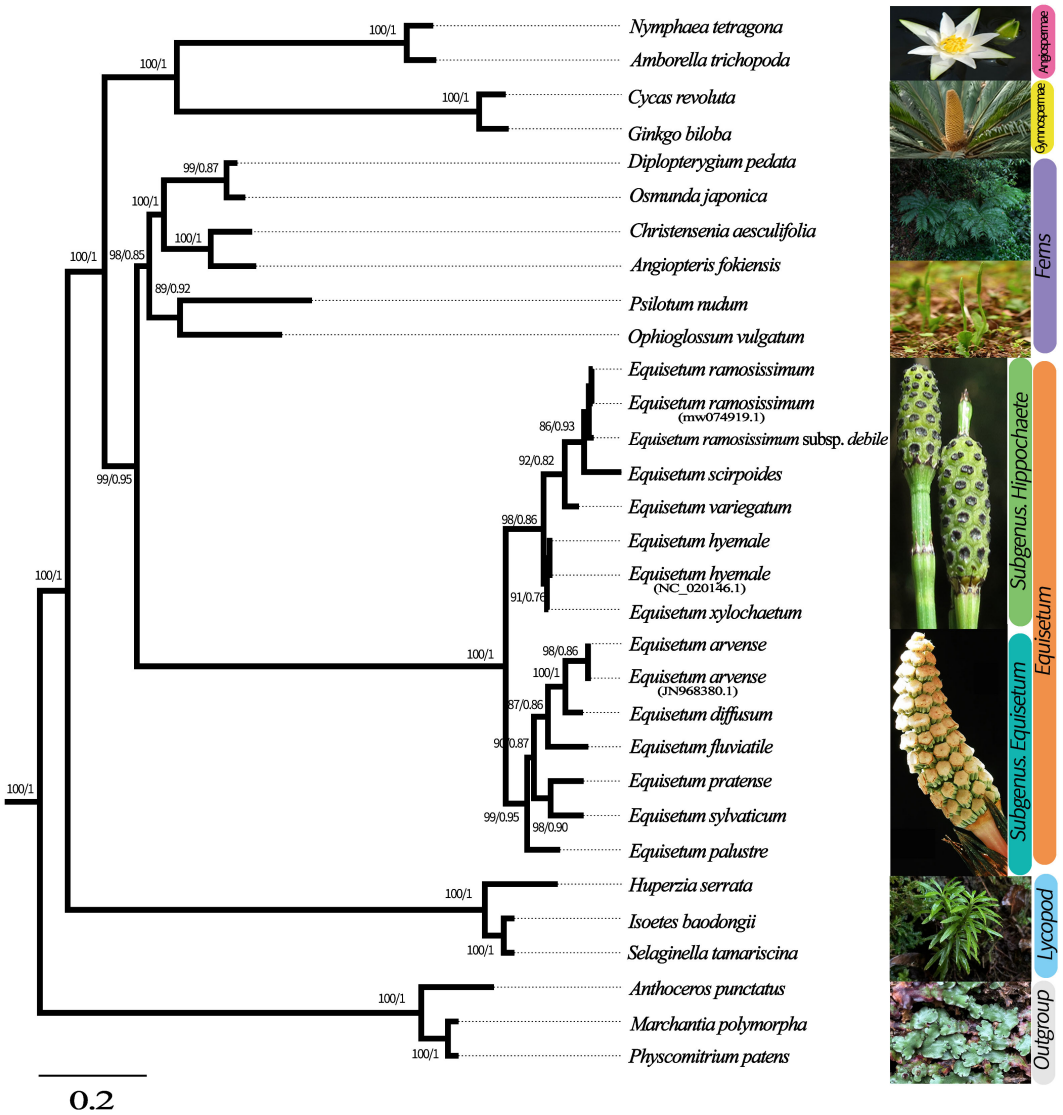
Phylogenetic tree based on Genes, CDSs and whole chloroplast genome concatenated analyses sequences from 28 species. The construction of phylogenetic trees was accomplished by employing both Bayesian inference (BI) and maximum likelihood (ML) methodologies. To present the level of support above the branches, we showcase Bayesian posterior probabilities (PP) and bootstrap percentages obtained from maximum likelihood analyses (BP).

To ascertain the phylogenetic connection between *Equisetum* species, we employed the total chloroplast genomes of 25 various species to reconstruct phylogenetic trees. *Physcomitrella patens*, *Anthoceros punctatus* and *Marchantia polymorpha* were used as outgroups in this analysis. **Supplementary Figures 4–6** shows the phylogenetic reconstruction obtained using BI and ML for the chloroplast genome, CDSs, and Gene dataset. Topologies of BI and ML were consistent in reconstruction of similar relationships with strong support in all analyses. The species tree obtained through ASTRAL shows the same topological structure with robust support (**Figure 3**). *Equisetum*, with strong support (PP =1.00, BP =100), formed a clade that was further divided into two prominent sub clades, supported by morphological features.

In the subgenus *Equisetum*, a clear distinction can be observed between *E. palustre* (supported by robust evidence, BP = 98, PP = 0.90) and the other species in the subgenus *Equisetum* (supported by strong evidence, BP = 100, PP = 1.00). Throughout the analyses, the relationship as sister species between *E. diffusum* and *E. arvense* consistently emerged, albeit with limited support (BP = 90, PP = 0.87), whereas the in *E. pratense* and *E. sylvaticum* formed a highly supported cluster (BP = 98, PP = 0.86). In subgenus *Hippochaete*, the subsequent clade exhibits *E. xylochaetum* and *E. hyemale* as sister species with robust support (BP =92, PP = 0.82). *E. ramosissimum* subsp*. debile* exhibits ambiguity in distinguishing it from *E. ramosissimum.*

### 3.3 Molecular clock analysis

**Figure 4:**
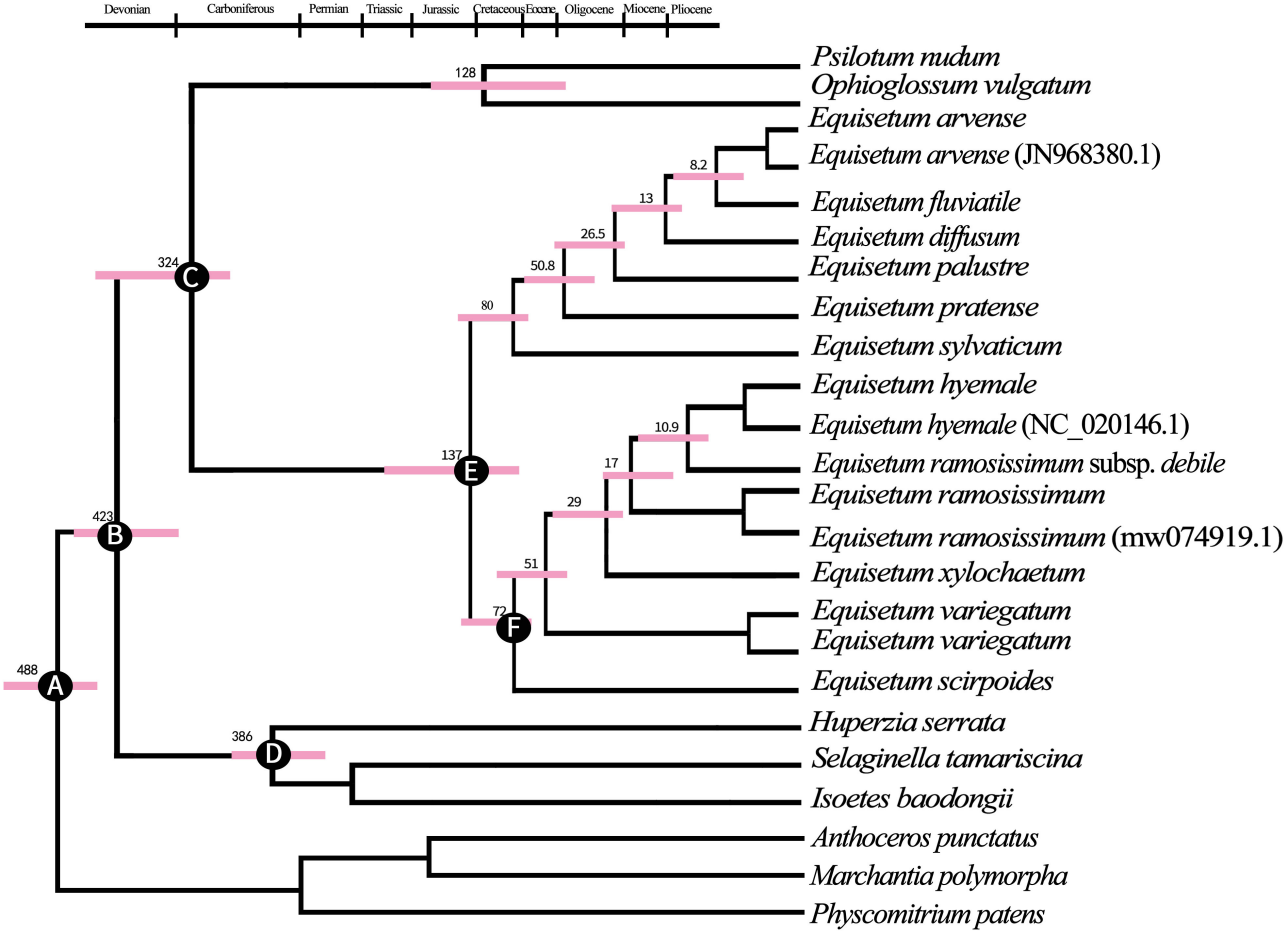
The maximum clade credibility tree of *Equisetum* was constructed using the BEAST method, based on the chloroplast genome sequences. This tree provided information on the mean ages and 95% highest posterior density (HPD) intervals for the node ages. **(A)** Secondary calibration 488.0 Mya **(B)** Fossil calibration 429.0 Mya **(C)** Fossil calibration 325.0 Mya **(D)** Fossil calibration 386.0 Mya **(E)** Fossil calibration 136.5 Mya **(F)** Fossil calibration 72 Mya.

According to the findings from BEAST analyses (**Figure 4**). The separation between Lycopods and ingroup Ferns most likely happened around 425 million years ago during the Devonian period (with a 95% highest posterior density of 323.6–426.8 million years ago). On the other hand, the separation between *Ophioglossaceae, Psilotaceae*, and *Equisetum* probably occurred around 324 million years ago during the Viséan age, which is part of the Middle Mississippian period in the Early Carboniferous era. During the Early Cretaceous period, the age at which all the existing *Equisetum* species originated was approximately 137 million years ago (Mya). This coincided with the common ancestor separation of subgenus *Hippochaete* and *Equisetum*. The coronal nodes of each subgenus are estimated to have formed during the Late Cretaceous period: around 80 Mya (ranging from 62.8 to 128.1 Mya) for subgenera *Equisetum* and about 72 Mya (ranging from 66.2 to 87.3 Mya) for subgenus *Hippochaete*. It was found that subgenus *Equisetum* exhibited earlier divergences among its major clades compared to subgenus *Hippochaete*. In particular, *Equisetum* underwent two splits before the Eocene era, while the primary divergences in *Hippochaete* took place from the Eocene to Miocene intervals. Nonetheless, it is likely that the most of variations among current *Equisetum* taxa originated quite recently, throughout the Middle to Late Miocene and extending into the Pliocene period.

### 3.4 Repeat sequence analysis and simple sequence repeats (SSRs)

**Figure 5:**
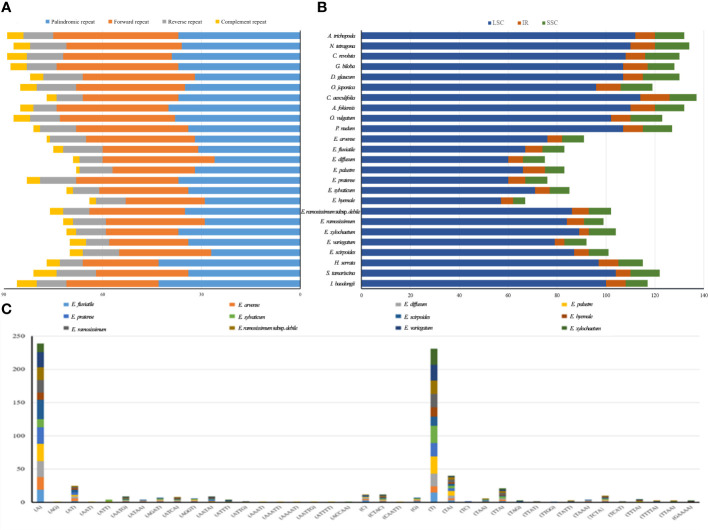
The classification and distribution of SSRs in the chloroplast genomes of *Equisetum*. **(A)** The difference of repeated sequence in chloroplast genomes. **(B)** Occurrence rate of SSRs. **(C)** Count of identified SSR motifs across various types of repeat classes.

In the chloroplast genomes, all anticipated lengthy patterns were retrieved, comprising 757 repeats as forward direction, 241 as reverse direction, 95 as complementary repeats, and 868 as palindromic repeats (**Figure 5A**). Among these patterns, *A. trichopoda* demonstrated the greatest count of long repeats, totaling to 90. In contrast, *E. hyemale* had the fewest long repeat sequences, with a count of 64. In order to explore the obvious genetic changes among *Equisetum* species, we conducted SSR analysis. Using the GMATA analysis, a comprehensive examination revealed that chloroplast genomes harbored a total of 2704 SSRs. As depicted in **Figure 5B**, the SSRs were primarily found in the LSC and SSC regions. Notably, with the number of SSRs in an individual species ranging from 67 (*E. hyemale*) to 134 (*N. tetragona*), with an average of 108 (**Figure 5C**). The significance of gene variation could potentially rely more on single nucleotide repeats compared to other forms of SSRs. Furthermore, only *E. ramosissimum* and *E. ramosissimum* subsp*. debile* chloroplast genomes revealed the presence of pentanucleotide and hexanucleotide repeats. The dominant SSRs in these genomes were single nucleotide repeats, indicating a bias towards A/T bases in the composition of SSRs.

### 3.5 The types of chloroplast genome mutations

**Figure 6:**
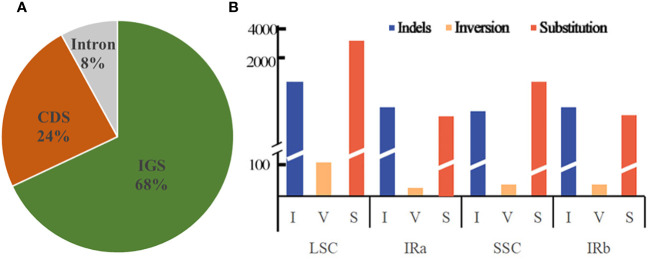
Forms of mutation in the *Equisetum* chloroplast genomes. **(A)** Location of the all indels from 12 species. **(B)** Number of indel, inversion and substitution sequences in the chloroplast genomes.

Dnasp v5.10 was employed to enumerate the frequencies of substitution, insertion/deletion, and inversion mutations that transpire in the chloroplast genome, of a dozen *Equisetum* species. The analysis disclosed a total of 6,492 occurrences of mutations within the chloroplast genome sequence of *Equisetum*. Among these, there were 1,953 instances of insertion/deletion, 49 occurrences of inversion, and 4,490 instances of substitution. The extent of the indels varied from 1 to 347 bp. Predominantly, the indels took place in IGS regions (constituting 68% of the events), while 24% occurred in CDS regions, and a mere 8% were observed in Intron regions (**Figure 6A**). A total of 4,985 mutations were found in the LSC region, including 1,523 insertion/deletion events, 36 inversion events and 3,426 substitution events. The mutation rate in the LSC region was 5.50%. A total of 646 mutations occurred in the IR region, including 196 insertion/deletion events, 8 inversion events and 442 substitution events. The mutation rate of the IR region was 1.32%. A total of 861 mutations occurred in the SSC region, including 234 insertion/deletion events, 5 inversion events and 622 substitution events. The mutation rate in the SSC region was 7.23% (**Figure 6B**).

### 3.6 IR contraction and expansion

**Figure 7:**
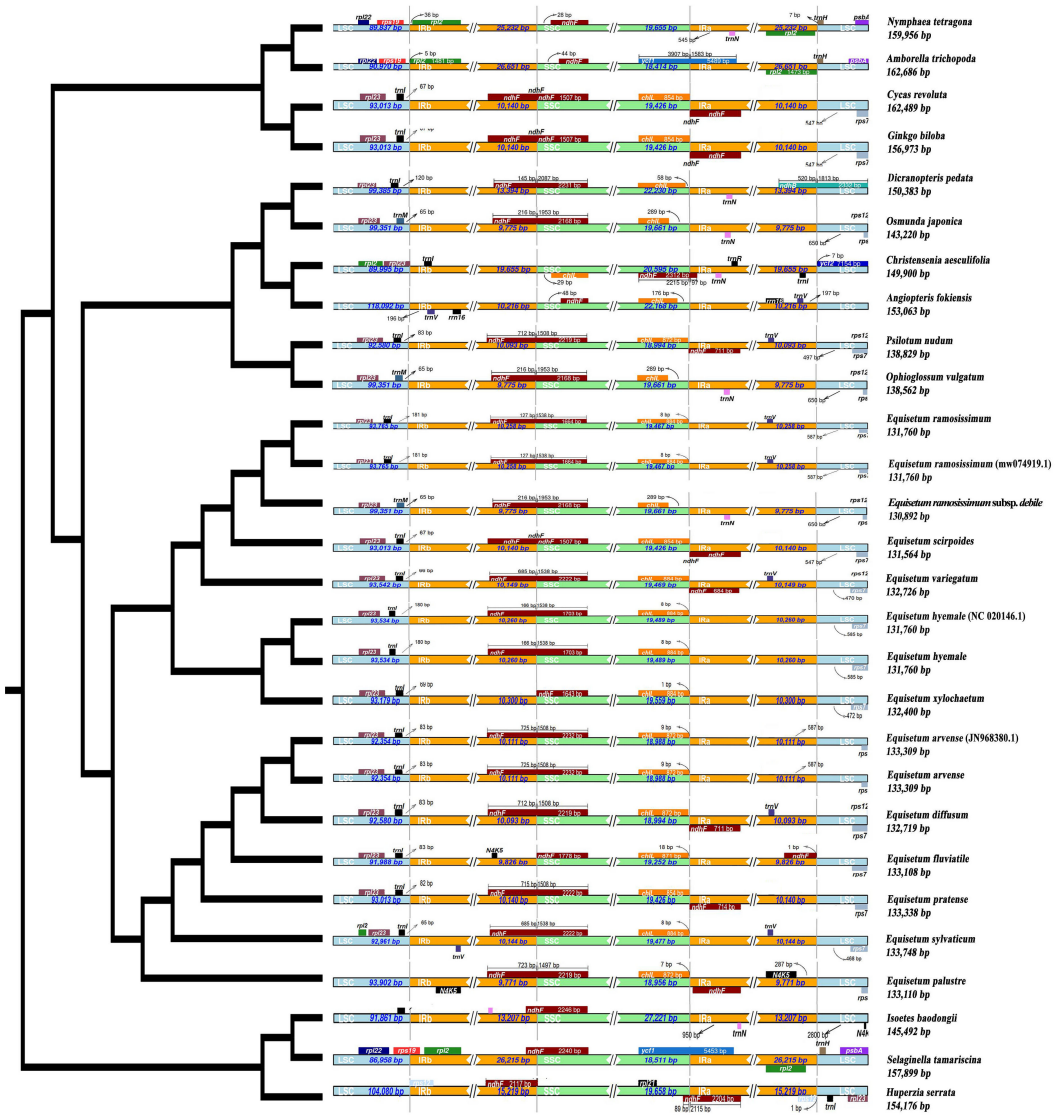
Depicts the comparison among the border regions of 25 distinct species chloroplast genomes. Compare the boundaries of large orders (LSC), small single copies (SSC), and the border between the reverse repetition (IR) region.

In this study, the variation of LSC, SSC and IR region boundaries of 25 chloroplast genomes was compared and analyzed with *Huperzia serrata* as a reference (**Figure 7**). Although the chloroplast genome size and structure of *Equisetum* species were highly conserved, the IR/SC boundary showed some differences among species due to the contraction/expansion of IR. The LSC/IRb boundary was slightly contracted. Excluding *E. xylosechaetum*, were found to have the *N4K5* and *rpl23* genes located at the junction of LSC/IRb. Additionally, the *trnL* gene was situated at a distance of 65-100 bp from the LSC/IRb boundary. In all species, the LSC/IRb of the chloroplast genome of *E. variegatum* exhibited the presence of the *rpl2* gene. The *ndhF* gene, with varying lengths from 1,507 to 2,232 bp, contained the IRb/SSC in all species. The truncation length of IRb was between 127 and 725 bp. In all *Equisetum* the *chlL* was found at the SSC region, precisely at the junction of SSC/IRa, with a distance of 1-18 bp from the SSC/IRa. But the *rpl21* was present in the SSC region of *Huperzia serrata*, it was absent in *Equisetum*. At the IRa/LSC of all *Equisetum*, the *rps7* was located at 464-565 bp of LSC.

### 3.7 Identification of variability hotspots

**Figure 8:**
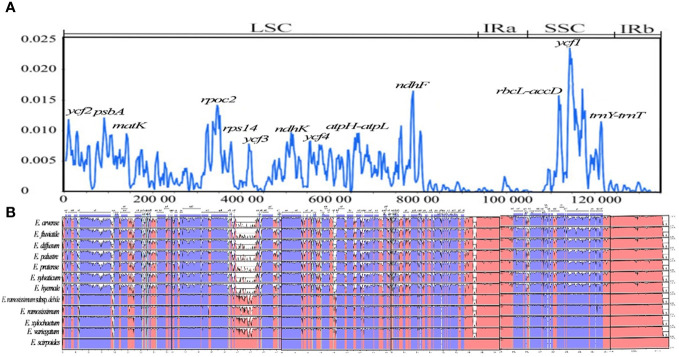
Visualization of comparison of *Equisetum* chloroplast genome sequences. **(A)** Assessment of nucleotide variability (*Pi*) within the chloroplast genome among 12 *Equisetum* species. **(B)** Color-coded representation to indicate genomic regions encompassing protein-coding sequences, rRNA, tRNA coding sequences, and conserved noncoding sequences (CNS). The vertical axis presents the percentage similarity, spanning from 50% to 100%.

By analyzing the chloroplast genomes of 12 distinct species, the mVISTA tool was utilized to identify regions of high variability, referencing *E. xylochaetum* as a reference. The findings revealed that while the gene order of the 12 *Equisetum* species remained largely consistent, there were significant differences in sequence composition among various regions including *ycf2, psbA, matK, rpoc2, rps14, ycf3, ndhK, ycf4, ndhF, ycf1, atpH-atpL, rbcL-accD, trnY(GUA)-trnT(GGU), ndhF-rpl32, trnY-trnE*, and *ndhC-trnV(UAC).* Notably, the non-coding sections displayed more pronounced dissimilarity in contrast to the protein-coding sections. The rRNA gene was highly conserved and there was almost no variation in it. In addition, compared with subgenus *Hippochaete*, subgenus *Equisetum* has more mutation sites, and the regions with relatively large variation are more likely to be distributed in the intergenic region. (**Figure 8B**).

In our investigation, we have assessed the average divergence of sequences between the 12 chloroplast genomes. The analysis conducted clearly demonstrates the extent of nucleotide variability (*Pi*) in these specific areas, ranging from 0.1% (*rpl32*) to 0.23% (*trnY-trnE*). By utilizing sliding window analysis, we have identified specific locations where mutations occur frequently. These mutational hotspots encompass *ycf2, psbA, matK, rpoc2, rps14, ycf3, ndhK, ycf4, ndhF, ycf1, atpH-atpL, rbcL-accD*, and *trnY (GUA)-trnT (GGU)*. Notably, these regions display higher *Pi* (>0.01) within the LSC and SSC. However, it is important to note that no single mutational hotspot was observed in the IR. The difference between the SSC and LSC areas exhibited a significant disparity in comparison to the IR regions, with the π value of the latter being notably reduced and showing a mirror-symmetrical pattern with the SSC at its core (**Figure 8A**).

### 3.8 Codon usage analyses

**Figure 9:**
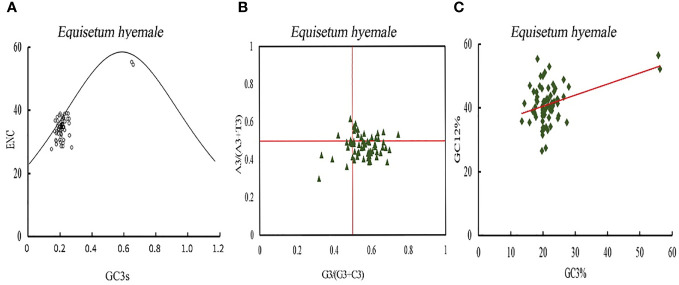
Codon bias analysis of chloroplast genomes of *Equisetum*. **(A)** ENC-plot analysis. **(B)** PR2-plot analysis. **(C)** Neutrality plot analysis.

The investigation of the primary factors that influence the development of codon usage inclinations is exhibited (**Figure 9**; **Supplementary Figure 2**). The real value of ENC for the majority of genes is below the anticipated value, signifying that the codon usage predisposition of protein coding genes in *Equisetum* is chiefly influenced by selective pressure, while only a few genes are affected by mutation. The assessment of bias in codon bases can mirror the disparity in the occurrence of A, T, C, and G bases. In the PR2-plot was generated distribution of genes in the four regions is not uniform vertically, with the majority of genes positioned below the midline. Horizontally, there is a higher number of genes on the right side of the midline compared to the left side (**Figure 9B**). This shows that in the third base the frequency of G position of chloroplast genome codon of *Equisetum* is greater than that of C, and the frequency of T is greater than that of A, which further indicates that most protein-coding genes in of *Equisetum* chloroplasts are affected by selection. Neutral mapping can be seen (**Figure 9C**) in that the distribution range of GC3 value is small (10% - 30%), the distribution range of GC12 value is large (20% - 60%), and Only a few genes are distributed along the line. The linear regression coefficients were between 0.28 and 0.46, indicating that mutation had the largest effect on codon usage preference. Therefore, codon usage bias in *Equisetum* chloroplasts is mainly affected by selection. The RSCU and △ RSCU values of plants in the *Equisetum* were calculated, and the results are shown in **Supplementary Figure 3**. The codons that exhibit high frequency and high expression were defined as the optimal codons for the chloroplast genome of the *Equisetum*. The optimal codons of plants in the *Equisetum* mostly end with A and U, with only one codon ending with G.

### 3.9 Selective pressure analysis

**Figure 10:**
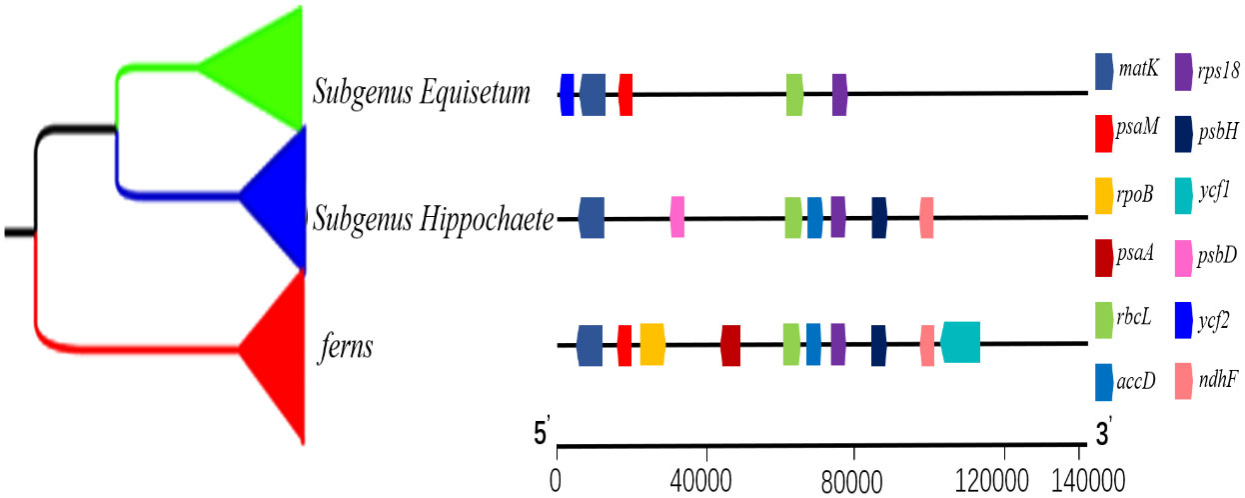
A Pairwise Ka/Ks ratios 12 *Equisetum* species. The genes of Ka/Ks > 1 from 12 chloroplast genome of *Equisetum.*.

To gain a deeper comprehension of the progression of plastid genes coding for proteins, an investigation was conducted employing *Equisetum* as the foreground branches while considering other ferns as the backdrop branches. The examination was specifically aimed at identifying the traces of natural selection using 78 protein coding genes. Notably, the Ka/Ks ratios were found to revolve around 0.2 in *Equisetum* species. Such outcomes imply that, in terms of the entirety of chloroplast proteins, *Equisetum* species have experienced more rigorous purifying selection compared to the background branches. Nine of the 78 genes were under selection (>1) (**Figure 10**; **Supplementary Table 3**), namely, 2 self-replication genes. The *rbcL* gene exhibits the highest number of loci that were positively selected. To differentiate, we employed subgenus *Equisetum* plants as our foreground branches, while subgenus *Hippochaete* served as the background branches. Among the five genes analyzed (*psaM, rps18, rbcL, matK*, and *ycf2*), exclusive sites showing positive selection occurred solely within the subgenus *Hippochaete*. We also performed the reverse analysis, using subgenus *Hippochaete* as foreground branches and subgenus *Equisetum* as background branches. Seven genes (*matK, psbD, rbcL, accD, psbH, ndhF* and *rps18*) contained positively selected sites unique within subgenus *Hippochaete*.

## 4 Discussion

### 4.1 Mutational dynamics and genetic diversity of *Equisetum* chloroplast genome

*Equisetum* chloroplast genomes displayed remarkable similarity of genome structure, genome order, genome content, and GC content. To gain a deeper understanding of sequence variation in *Equisetum*, this study identified crucial types of genetic variation in chloroplast genomes, such as nucleotide substitutions, repeats, and indels. Alongside nucleotide substitutions, a total of 2, 704 SSRs, 868 long repeats, and 248 indels were also detected. Plants commonly experience the enlargement and reduction of their chloroplast genome as part of their evolutionary process. The IR regions of *E. xylochaetum* were discovered to possess the smallest size among all *Equisetum* chloroplast genomes. Moreover, an extensive analysis revealed that the SC/IR regions of *E. xylochaetum* exhibited the highest frequency of sequence deletions, implying a potential link between deletions and IR region contraction. Conversely, the LSC region of *E. xylochaetum* exhibited remarkable elongation in comparison to other regions. Notably, our investigation uncovered a substantial presence of repetitive sequences in the LSC/IR area, underscoring the impact of repeats on the overall size of the chloroplast genome. Hence, it can be deduced that the smaller of the *Equisetum* chloroplast genome, as compared to ferns, is a result of the influence exerted by repeats and indels on its structural configuration.

Mutation events in the plastid genome are generally randomly distributed. Both eukaryotic and prokaryotic genomes share a remarkable characteristic, which is the simultaneous existence of nucleotide substitutions and indel mutations (Ahmed et al., 2012). Repeat sequences and indel sequences were found to be closely associated with the regions in *Equisetum* chloroplast genomes that displayed discrepancies. Meaningful correlations were observed in the comparisons made among the three mutation categories, further supporting the theories proposing that mutations are caused by indels and repeats. According to the hypothesis concerning indel-induced mutations, DNA repair is initiated in response to indels, ultimately leading to genetic alterations (Tian et al., 2008). Contrary to this, the hypothesis of repeat-induced mutation proposes that mutations arise from the presence of repetitive sequences instead of indels alone. These repetitive sequences facilitate the halt of replication-fork, leading to the enlistment of error-prone polymerases. As a consequence, this gives rise to the emergence of indels and nucleotide substitution (McDonald et al., 2011). According to our findings the model of indels and substitutions showed the strongest correlation and highest level of statistical significance (**Supplementary Table 3**). Therefore, we believe that these two hypotheses are not mutually exclusive in the evolution of the *Equisetum* chloroplast genome, but rather interact to jointly promote the evolution of the *Equisetum* chloroplast genome. Additionally, the findings implied that through the identification of distributed repetitive or inserted sequences, it might become feasible to anticipate regions characterized by substantial mutation frequencies and ascertain particular sequences conducive to genetic analysis, contingent upon their relative locations.

### 4.2 Signature of positive selection on chloroplast genome driving adaptation evolution

Notably, in our analysis of the GC content in chloroplast genomes of *Equisetum* species in comparison to fern species, we observed that *Equisetum* had the lowest total GC content (<33.9%) (**Supplementary Figure 1**). G/C base pairs have three hydrogen bonds, which are more stable compared to the two hydrogen bonds of A/T base pairs (Jia et al., 2010). The tRNA and rRNA sequences were considerably rich in GC bases; as a consequence, the IR regions rich in rRNA and tRNA appear to have had higher GC content than the other regions; high GC content in the IR regions rich in rRNA and tRNA appear may drive its stability compared to the LSC and SSC regions. Significant variations in GC content are observed by analyzing the chloroplast genomes of closely related species inhabiting diverse environments. This variability directly affects the amino acid composition of proteins in their specific habitats (Foerstner et al., 2005). Thus, we propose the hypothesis that genes exhibiting lower GC content are more susceptible to transcription compared to those with higher GC content. *Equisetum* has a lower GC content might be explained by the high level of RNA editing in the organelles (Smith, 2009). It has been reported that fern chloroplast genomes have evolved a higher number of RNA editing events than spermatophyte chloroplast genomes. It has been reported that the high number of C-to-U conversions developed in the early stages of vascular plant evolution (Koichiro et al., 2008). Consequentially, this phenomenon can potentially be elucidated by natural selection it can be inferred that the selective pressure exerted by the distinctive environment inhabited by *Equisetum* species (damp and dark) has contributed to the overall decrease in GC content within their chloroplast genomes.

The bias of the codon usage in the plant cp genome was an important evolutionary feature for the studies regarding mRNA translation, new gene discovery, and molecular biology (Yang et al., 2014). Previous studies have confirmed that genes tend to choose preferred synonymous codons for specific amino acids rather than randomly distributions (Sorimachi, 2010; Li et al., 2019). Our study showed that genes of *Equisetum* prefer codons with A/T in the third position, which was consistent with previous studies. There is a certain difference between the actual value of ENC and the expected value of ENC, indicating that natural selection has a significant impact on codons: the uneven distribution of coding genes is used with frequencies T>A, G>C; The main factor affecting the formation of codon preference is natural selection, which is similar to the results of *Arabidopsis thaliana* and *Oryza sativa* (Jia and Xue, 2009). It is inferred that the codon preference characteristics of the chloroplast genome of the *Equisetum* genus are more affected by natural selection pressure than other factors. The greater the positive selection and mutation pressure, the more optimal codons are formed in the genome; On the contrary, the greater the negative selection effect, the less optimal codons there are. Based on the RSCU values of the codons in the chloroplast genome of the *Equisetum* genus, this study identified only 13 common optimal codons, suggesting that their chloroplast genome codon usage preferences are within the range of purification selection. In the process of protein translation, the optimal codon can effectively improve its speed and accuracy, and the preference for the optimal number of codons is mainly influenced by strong positive selection (Paul and Malakar, 2018; Li et al., 2021).

*Equisetum* are present in widely distributed around the world and within heterogeneous ecological niches (Harrison, 2005). This suggests the occurrence of adaptive radiation, which may have left a selection footprint on the chloroplast genome. During our analysis were subjected to positive selection of 12 plastid genes (*rpoB, ycf1, psaA, psaM, psbH, psbD, rbcL, ndhF, matK, accD, rps18* and *ycf2*), indicating that they may have contributed to environmental adaptation in *Equisetum*. Remarkably, the photosynthesis-related gene *rbcL* encoding the RuBisco’s large subunit, which displayed particularly pronounced indications, is commonly under positive selection in terrestrial plants but not in water plant (Li et al., 2022), possibly due to more unstable thermal regimes on land (Kapralov and Filatov, 2007). In this study, we collected *Equisetum* from habitats with diverse sunlight exposure, and thus *rbcL* may be probably in response to adaptive evolution associated with sunlight and thermal conditions. An extremely significant positive selection site at the loci in *matK* of *Equisetum*. The gene *matK* is transcribed from the sole intact plastid group intron ORF localized, *matK* has lost do mains assigned to a reverse transcriptase and endonuclease function (Liere and Link, 1995; Wicke et al., 2011). The *ndhF* gene has been proposed to regulate the redox state, thereby maintaining or enhancing the photosynthetic efficiency of certain crops when subjected to extreme temperatures and changes in light intensity (Martín et al., 1787). Consequently, positive selection acting upon this gene may have contributed to the acclimatization to various environmental stressors (like reduced levels of CO_2_ partial pressure) and shifts in climate conditions that *Equisetum* has experienced (Hermida-Carrera et al., 2017; Jiang et al., 2018; Yao et al., 2019). Due to the extremely high Ka/Ks value of *ycf2* gene, it is a valuable resource for future research on the adaptive evolution of *Equisetum*. The *ycf2* has become a useful gene for asses sing sequence variation and evolution in plants (Huang et al., 2010). *Psa* and *psb* genes are primary members of photosystem which may evolve rapidly in some *Equisetum* species. Additionally, there is proof indicating that certain subgenus *Equisetum* species have varying levels of transpiration, with *E. fluviatile*, the most water-loving species, exhibiting the greatest rates of transpiration (Spatz et al., 1998). In the subgenus *Equisetum*, stomata are located at the identical level as the epidermal surface, whereas in the subgenus *Hippochaete*, stomata are positioned beneath the epidermal surface. Considering the close correlation between hydraulic conductivity and stomatal conductance (Marsh et al., 2000), it is probable that hydraulic “bottlenecks” exist at the nodes of *Equisetum* stems. These bottlenecks restrict water transport, ultimately leading to a decrease in stomatal conductance. The positive selection genes also play crucial roles in chloroplast protein synthesis, energy transformation and regulation, and photosynthesis. These results indicate the diverse adaptive evolution in plastid genes of *Equisetum*. Among the photosynthesis related genes (*ndhF, rbcL, ycf1*, and *ycf2*) contained positively selected sites unique in most vascular plants. In addition, *accD, ycf1* and *ycf2* had the most accelerated rates as detected in plastomes of *Dryopteris goeringiana* and *Dryopteris crassirhizoma* (Fan et al., 2021). The positive selection in the five genes probably help *Equisetum* efficiently capture light energy to produce adequate nutrition to adapt to their growth and development under extreme and variable environmental conditions.

### 4.3 The divergence hotspot regions and candidate DNA barcoding of *Equisetum*

The universal DNA barcodes have lower divergence and poor discriminatory power. The mutation events in the chloroplast genome are not universally randomly distributed within the sequence and are concentrated in certain regions forming the “hotspot” regions (Dong et al., 2012). Divergence hotpots in chloroplast genomes have been widely utilized for delimitation of closely related species of plants (Bi et al., 2018; Dong et al., 2021). The highly variable hotspots regions identified have potential as candidate markers or DNA barcodes for inferring the phylogeny of *Equisetum*. The cp genomes of *Equisetum* species are generally consistent in overall gene content and arrangement. However, comparative genome analysis using mVISTA and nucleotide variability (*Pi*) revealed relatively conserved sequences among *Equisetum* species. Notably, the variability observed in the IR exhibited considerably less disparity compared to that in the SSC and LSC within the *Equisetum* chloroplast genome. All regions were found in the single-copy and intergenic regions. As observed in other vascular plant, the IR and coding regions exhibited lower levels of divergence than the single copy and non-coding regions (Lu et al., 2016; Yin et al., 2018; Wu et al., 2021). This result was consistent with that in fern plastomes (Wei et al., 2017).

This study identified divergence hotspot regions (*ycf2, psbA, matK, rpoc2, rps14, ycf3, ndhK, ycf4, ndhF, ycf1, atpH, atpL, rbcL, accD*, and *trnY (GUA)-trnT (GGU)*) within the chloroplast genome of *Equisetum*. *MatK* is a useful biomarker for phylogenetic analysis in plant classification because its sequence evolution is faster than that of other chloroplast genes (Selvaraj et al., 2008). *AccD* IGS and *ycf1* CDS stood out as the most distinct areas when considering their uniqueness. These two regions encompassed the largest tandem repeats (TRs) and served as the hypervariable regions and arrangement endpoints of *Equisetum*. We examined the characteristics of the *ycf1* gene across species of *Equisetum*. The *ycf1* genes exhibited a remarkable increase in length, with widespread insertions of TRs. Potential rearrangement endpoints were also their location. The *accD* gene and the surrounding sequences exhibited significant expansion as a result of TR insertion. Furthermore, in *Equisetum* the arrangement of its surrounding genes and the insertion position of TR on *accD* exhibited significant dissimilarities. Consequently, it would be more appropriate to employ the *ycf1* and *accD* gene region as promising candidates for the DNA barcodes used in the classification and phylogeny analysis of the *Equisetum*. The highly mutable *accD* gene sequence, which was also utilized as a potential DNA barcode of Quercus (Liu et al., 2019), and the ycf1 gene, reportedly serving as a potential DNA barcode in the Papaveraceae (Parks et al., 2018), were identified. Additionally, Liu et al. (2017) discovered that *ycf1* can as a distinctive DNA barcode for *Equisetum*. However, this marker was not extensively used in plant phylogeny and DNA barcoding.

### 4.4 Phylogenetic analysis and divergence time in *Equisetum*

Previous research investigating the phylogeny of the *Equisetum* genus, with the utilization of a limited number of markers derived from nuclear and plastid DNA, have reported instances where the relationships among species within certain clades of the *Equisetum* genus diverge between chloroplast and nuclear gene markers (Kenrick and Crane, 1997). The insufficiency of the phylogenetic method’s limited number of markers in determining *Equisetum* relationships necessitates the inclusion of more plastid genomes and additional molecular information from other genetic compartments. This study is the first to provide the phylogenetic backbone for *Equisetum* using chloroplast genomes. The presented phylogenetic relationships here validate the monophyly of *Equisetum* and demonstrate a distinct division of the genus into subgenera (*Equisetum* and *Hippochaete*). This finding aligns with previous investigations on morphology and molecular analyses employing the supermatrix comprising of four plastid markers (Christenhusz et al., 2019). Among published analyses to date, *Equisetum* has been inconsistent in position. However, given that *Equisetum* has been shown to be sister to all other ferns in some analyses (Testo and Sundue, 2016; Shen et al., 2018). This is consistent with our research findings *Equisetum* based on four datasets (chloroplast whole genome, chloroplast genome shared CDSs dataset, Gene and chloroplast whole genome, CDSs and Gene concatenated analysis). Intended to explore and compare the effects of molecular markers based on different genetic backgrounds on phylogenetic structures, in order to explore more authentic and comprehensive interspecific relationships. The phylogenetic trees constructed in all datasets have clearly displayed the phylogenetic relationships, with consistent topological structures across the larger evolutionary branches and high support rates. According to the topological structures, the generalized pteridophyte can be divided into Lycophytes, Equisetaceae and ferns. The *Equisetum* can be clearly divided into two large evolutionary branches subgenus *Hippochaete* and *Equisetum*, and is supported by morphological features.

Here, the comparative genomic analyses have revealed that *Equisetum* contained diverse genomic variations. The phylogenomic study showed that Equisetaceae was monophyletic. Our molecular clock estimates of divergence place the origin of the *Equisetum* lineage in the Early Carboniferous (~342 Mya). *Equisetum* is clearly one of the most evolutionarily isolated and possibly the oldest extant vascular plant genus (Husby, 2013; Vanneste et al., 2015). Molecular dating indicated that the emergence of *Equisetum* occurred around 137 million years ago (Mya), as supported by a 95% highest posterior density (HPD) interval ranging from 95.8 to 177.0 Mya. The ferns originated approximately 128 Mya, and molecular dating confirmed that *Equisetum* is sister all ferns. Shen et al. (2018) conducted an extensive analysis using transcriptome sequencing data, which yielded results that are overall consistent with our findings. The subgenera of *Equisetum*, despite being sister taxa, exhibit considerable genetic divergence (**Figure 3**; **Supplementary Figures 4–6**). Moreover, distinctive dissimilarities in both morphology and ecological preferences exist, underscoring their recognition at the subgenus level. The crown group age of subgenus *Equisetum* was approximately 80 Mya (95% HPD, 62.8-128.1 Mya) and that for subgenus *Hippochaete* was approximately 72 Mya (95% HPD, 66.2-87.3 Mya) (**Figure 4**). The timing obtained for the division of the main clades closely corresponds to the results of Zamaloa et al. (2022), who solely relied on the ages of the fossil species incorporated to ascertain the time of divergence.

The results support that Equisetaceae and ferns are sister groups, and the chloroplast genomes of Equisetaceae and ferns have very low similarity with the chloroplast genomes of ferns. Through comparison, it is found that the similarity of chloroplast genomes of Equisetaceae and ferns is as low as about 57%, and the chloroplast genomes of the two are significantly different in terms of gene number and gene order, indicating that the chloroplast genomes of the two have different evolutionary patterns. Combined with the phylogenetic analysis of chloroplast genomes, it is supported that Equisetaceae and ferns are sister groups.

## 5 Conclusions

Twelve different *Equisetum* species and thirteen other vascular plants species analyzed in this study. Based on the findings, it was observed that *Equisetum* chloroplast genomes exhibited analogous fundamental structure, size, gene number, order, and functional arrangement. However, it is worth noting that *Equisetum* chloroplast genomes demonstrated a comparatively lower GC content in contrast to its fern counterparts. Additionally, we observed the identification of highly variable nucleotide hotspots within mutational regions, indicating the potential use of the *ycf1* and *accD* genes as species identification and taxonomy markers. The presence of inverted repeat regions and simple sequence repeats regions contributes to significant variations in genome size among divergent genes. In addition to the presence of highly diverse genes, repeat and indel-induced mutations contribute significantly to the diversity of *Eqiusetum* chloroplast genomes. Utilizing the complete cp genomes dataset, we constructed an evolutionary tree and conducted molecular dating analyses. These evaluations not only emphasized the considerable phylogenetic divergence between *Equisetum* and ferns but also provided robust evidence supporting the designation of the pteridophyte are divided into Lycophytes, Equisetaceae and ferns. In summary, our investigation presents a novel framework that enhances comprehension of species delimitation, genome evolution, and phylogenetic associations within *Equisetum*.

## Data availability statement

The data presented in the study are deposited in the GenBank repository, accession number PP501410-PP501411.

## Author contributions

WS: Data curation, Formal analysis, Software, Validation, Visualization, Writing – original draft, Writing – review & editing. ZW: Validation, Visualization, Writing – review & editing. YG: Investigation, Writing – review & editing. TW: Investigation, Writing – review & editing. BL: Project administration, Supervision, Writing – review & editing. YY: Funding acquisition, Project administration, Supervision, Validation, Writing – review & editing.
