# Supplementary material for: Chloroplast genome structure analysis of Equisetum unveils phylogenetic relationships to ferns and mutational hotspot region
Source: Front Plant Sci. 2024 Apr 11;15:1328080. doi: 10.3389/fpls.2024.1328080 (PMC11044155; doi:10.3389/fpls.2024.1328080)
Supplement: Supplementary file 1 [file DataSheet_1.docx]

Supplementary Material

# Supplementary Figures and Tables

##
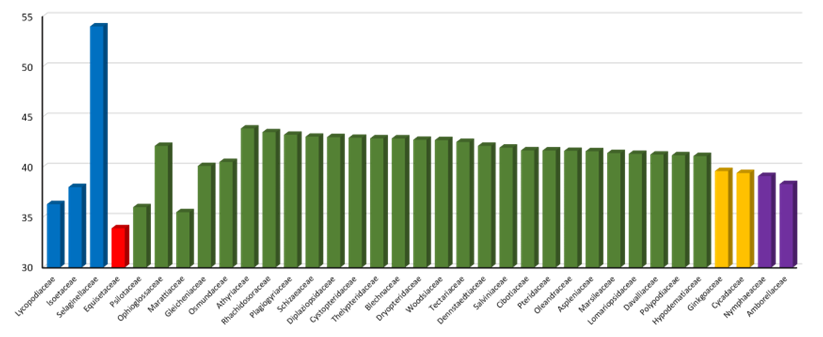
Supplementary Figures

## Supplementary Figure 1. Distribution of GC content in chloroplast genomes of vascular plants.


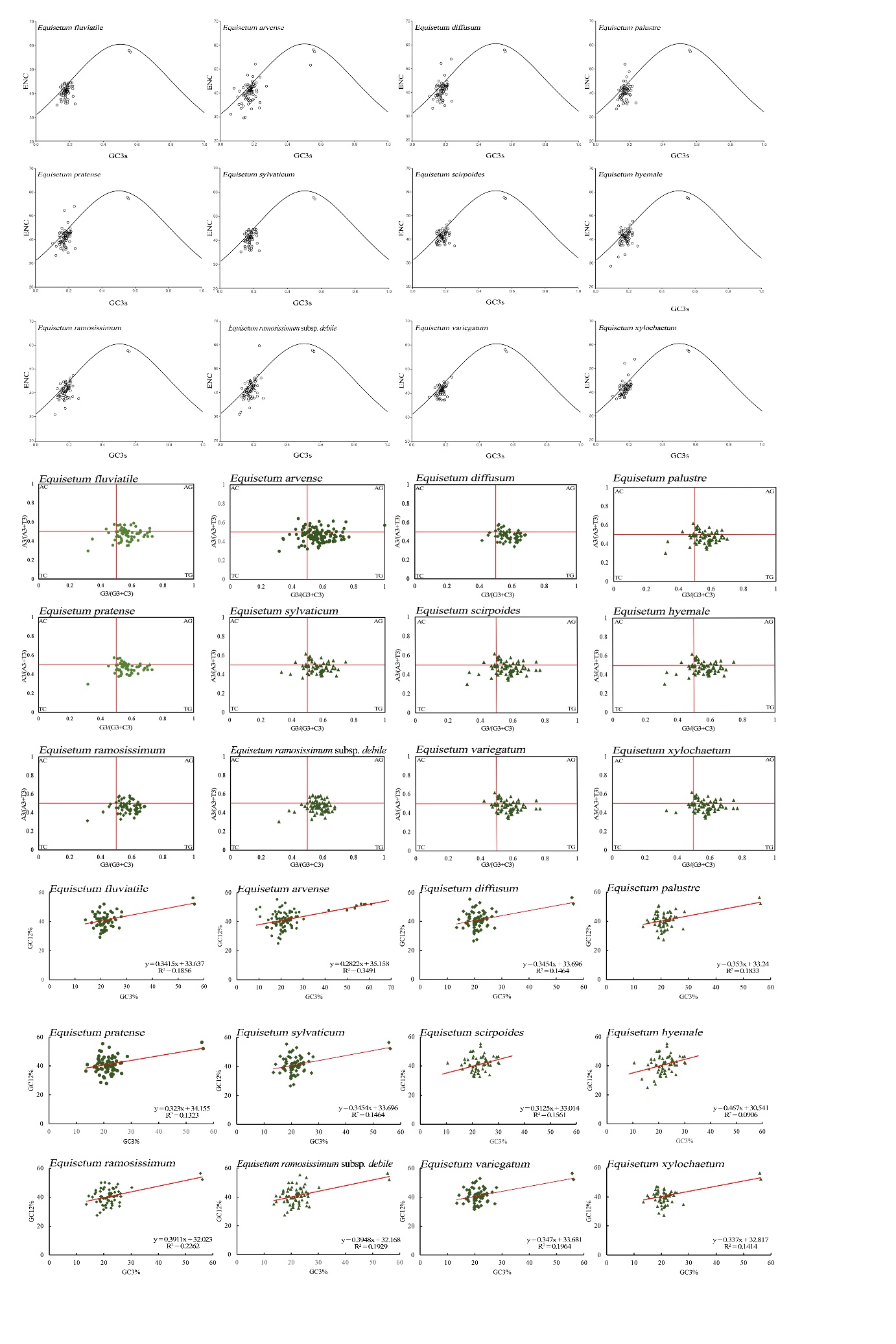


**Supplementary Figure 2.** Codon bias analysis of chloroplast genomes of 12 species of *Equisetum*.

**
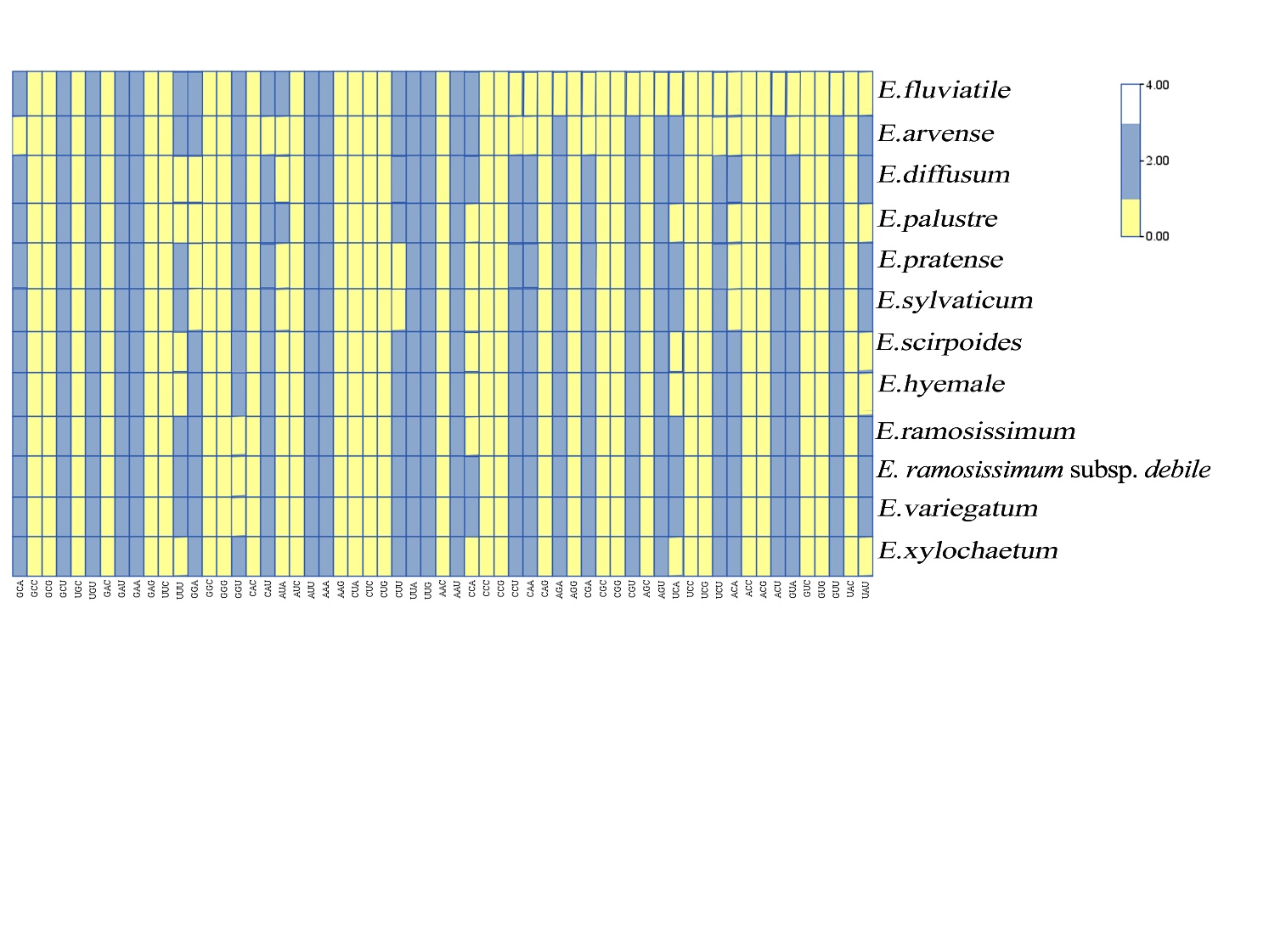
**

**Supplementary Figure 3.** Optimal codons analysis in the chloroplast genome of *Equisetum.*

**
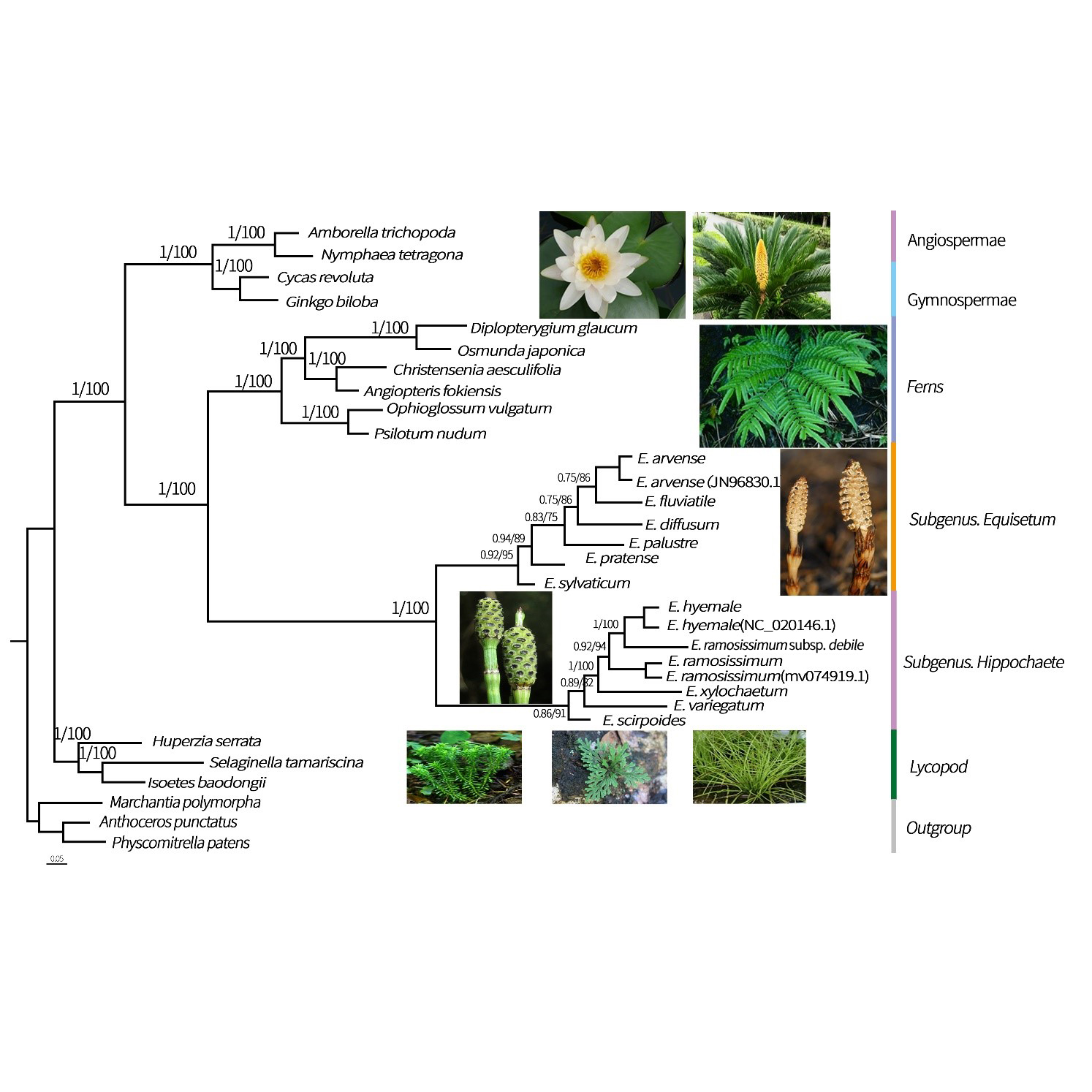
**

**Supplementary Figure 4.** The construction of phylogenetic trees based on the chloroplast genome. To present the level of support above the branches, we showcase Bayesian posterior probabilities (PP) and bootstrap percentages obtained from maximum likelihood analyses (BP).


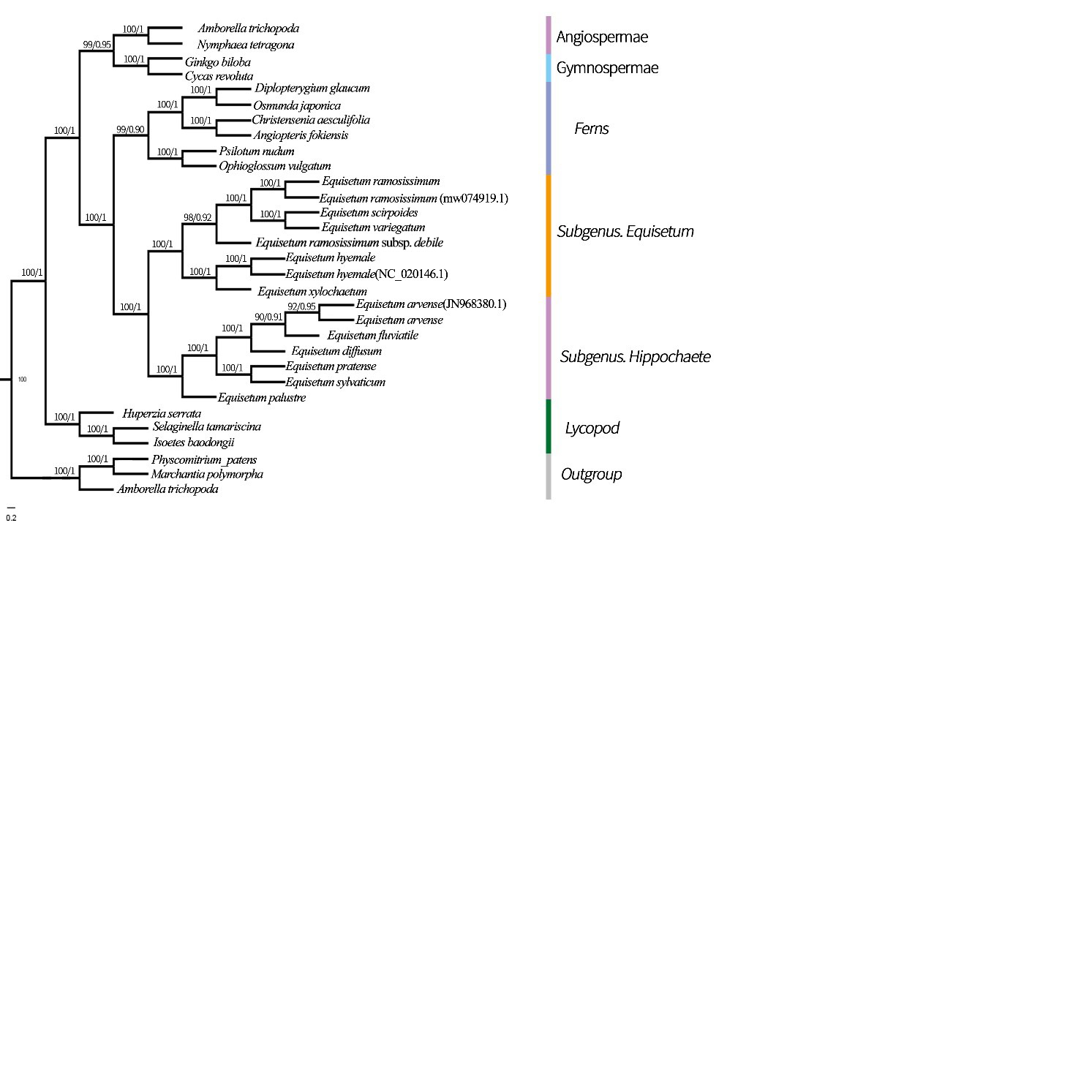


**Supplementary Figure 5.** The construction of phylogenetic trees based on the CDSs. To present the level of support above the branches, we showcase Bayesian posterior probabilities (PP) and bootstrap percentages obtained from maximum likelihood analyses (BP).


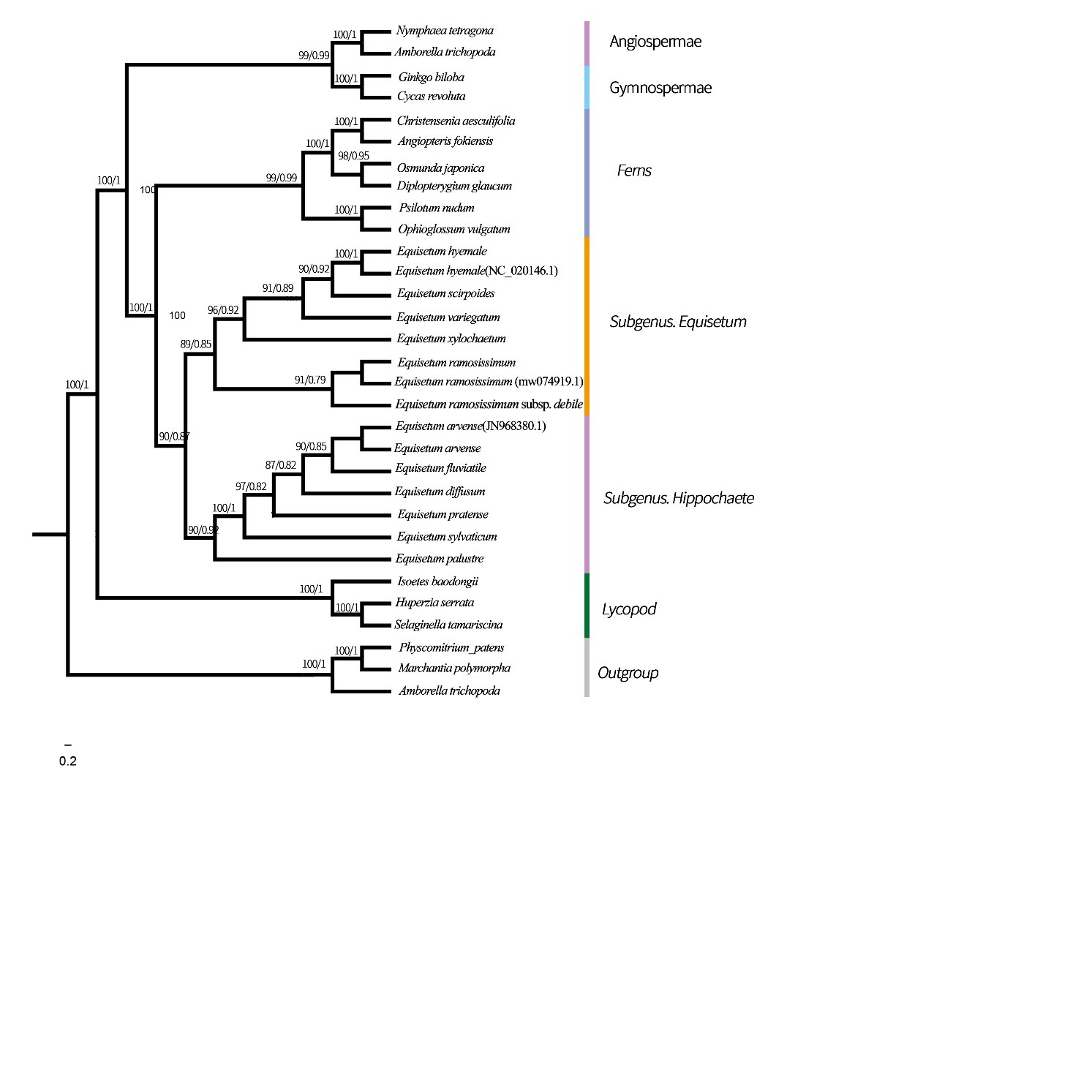


**Supplementary Figure 6.** The construction of phylogenetic trees based on the Gene. To present the level of support above the branches, we showcase Bayesian posterior probabilities (PP) and bootstrap percentages obtained from maximum likelihood analyses (BP).

## Supplementary Table

## Supplementary Table S1． Information of all the employed samples, * were new sequencing for this study

| No. | Family name | Species | Locality |
| --- | --- | --- | --- |
| *YYH23766 | Equisetaceae | *E. fluviatile* | Yichun, Heilongjiang, China |
| *YYH23564 | Equisetaceae | *E. arvense* | Haerbin, Heilongjiang, China |
| *YYH23791 | Equisetaceae | *E. diffusum* | Kunming, Yunnan, China |
| *YYH23787 | Equisetaceae | *E. palustre* | Xilinguole, Neimenggu, China |
| *YYH23792 | Equisetaceae | *E. pratense* | Haerbin, Heilongjiang, China |
| *YYH25539 | Equisetaceae | *E. sylvaticum* | Shangzhi, Heilongjiang, China |
| *YYH60538 | Equisetaceae | *E. scirpoides* | Mohe, Heilongjiang, China |
| *YYH23383 | Equisetaceae | *E. hyemale* | Haerbin, Heilongjiang, China |
| *YYH23781 | Equisetaceae | *E. ramosissimum* | Kunming, Yunnan, China |
| *YYH23450 | Equisetaceae | *E. ramosissimum* subsp*. debile* | Shenzhen, Guangdong, China |
| *YYH24578 | Equisetaceae | *E. variegatum* | Daqing, Heilongjiang, China |
| MW282958 | Equisetaceae | *E. xylochaetum* | Atacama Desert, Chile |
| OP831286.1 | lsoetaceae | *I. baodongii* | Zhuji, Zhejiang, China |
| NC_033874.1 | Lycopodiaceae | *H. serrata* | Helong, Jilin, China |
| MN894555 | Selaginellaceae | *S. tamariscina* | Republic of Korea |
| AP004638.1 | Psilotaceae | *P. nudum* | Gofuku, Toyama, Japan |
| MZ066610.1 | Ophioglossaceae | *O. vulgatum* | Tianhe, Guangzhou, China |
| NC_068854.1 | Marattiaceae | *A. fokiensis* | Nanning, Guanxi, China |
| MN412587.1 | Marattiaceae | *C. aesculifolia* | Valencia, California, USA |
| MN817664 | Gleicheniaceae | *D. pedata* | Kunming, Yunnan, China |
| MK554796 | Osmundaceae | *O. japonica* | Dalian, Liaoning, China |
| OP105160.1 | Ginkgoaceae | *G. biloba* | Nanjing, Jiangsu, China |
| NC_020319.1 | Cycadaceae | *C. revoluta* | Beijing, China |
| NC_057565.1 | Nymphaeaceae | *N. tetragona* | Zhenjiang, Jiangsu, China |
| NC_005086.1 | Amborellaceae | *A. trichopoda* | Jena, Germany |
| LC516510 | Funariaceae | *P. patens* | Kyoto, Japan |
| NC_037507 | Marchantiaceae | *M.polymorpha* | Melbourne, Australia |
| MN544310 | Anthocerotaceae | *A.punctatus* | New York, USA |

# Supplementary Table S2． Genes contained in the *Equisetum* chloroplast genome

| **Group of gene** | **Name of gene** |
| --- | --- |
| **Photosynthesis-related genes** |  |
| Rubisco | *rbcL* |
| Photosystem I | *psaA psaB psaC psaI psaJ*  *psaM* |
| Assembly and stability of photosystem I | *ycf3 ycf4 ycf12* |
| Photosystem II | *psbA psbB psbC psbD psbE  psbF psbH psbI psbJ psbK  psbL psbN psbM psbT psbZ* |
| ATP synthase | *atpA atpB atpE atpF atpH  atpI* |
| Cytochrome b/f complex | *petA petB petD petG petL*  *petN* |
| Cytochrome c synthesis | *ccsA* |
| **Transcription- and translation-related genes** | |
| Transcription | *rpoA rpoB rpoC1 rpoC2* |
| Small ribosomal subunit | *rps2 rps3 rps4 rps7 rps8  rps11 rps12 rps14 rps15 rps18*  *rps19* |
| Large ribosomal subunit | *rpl12 rpl16 rpl14 rpl20 rpl21*  *rpl22 rpl23 rpl32 rpl33 rpl36* |
| Translation initiation factor | *infA* |
| **RNA genes** |  |
| Ribosomal RNA | *rrn5* rrn4.5* rrn16* rrn23** |
| Transfer RNA | *trnV-GAC* trnI-GAU* trnA-UGC* trnR-ACG* trnN-GUU* trnL-UAG trnP-GGG trnI-CAU trnP-UGG trnW-CCA  trnR-CCG trnM-CAU trnV-UAC trnF-GAA trnL-UAA  trnT-UGU trnS-GGA trnfM-CAU trnT-GGU trnS-UGA  trnG-GCC trnE-UUC trnY-GUA trnD-GUC trnC-GCA  trnR-UCU trnG-UCC trnS-GCU trnS-CGA trnQ-UUG  trnK-UUU trnH-GUG trnL-CAA* |
| **Other genes** |  |
| RNA processing | *matK* |
| Carbon metabolism | *cemA* |
| Fatty acid synthesis | *accD* |
| Proteolysis | *clpP* |
| **Genes of unknown function** |  |
| Conserved reading frames | *ycf1 ycf2* |
| NADH dehydrogenase | *ndhA ndhB ndhC ndhD ndhE*  *ndhF ndhG ndhH ndhI ndhJ*  *ndhK* |

# Supplementary Table S3 Correlation analysis of three types of mutation

# Supplementary Table S4 Maximum likelihood parameter estimates and substitutions for the12 plastid genes in *Equisetum* (*rpoB, rps18, psaA, psaM, psbH, psbD, rbcL, ndhF, matK, accD, ycf1 and ycf2*).

| ***Plstid gene*** | | **Species 1** | **Species 2** | **Ka** | **Ks** | **Ka/Ks** |
| --- | --- | --- | --- | --- | --- | --- |
| *matK* | *Equisetum fluviatile* | | *Equisetum arvense* | 0.00172662 | 0.00153603 | 1.1240796 |
| *matK* | *Equisetum fluviatile* | | *Equisetum diffusum* | 0.00172662 | 0.00151603 | 1.1389089 |
| *matK* | *Equisetum fluviatile* | | *Equisetum palustre* | 0.00178323 | 0.00161862 | 1.1016977 |
| *matK* | *Equisetum fluviatile* | | *Equisetum pratense* | 0.00320983 | 0.00246146 | 1.304035 |
| *matK* | *Equisetum fluviatile* | | *Equisetum sylvaticum* | 0.00320983 | 0.0030146 | 1.0647615 |
| *matK* | *Equisetum fluviatile* | | *Equisetum scirpoides* | 0.00270983 | 0.00246146 | 1.1009035 |
| *matK* | *Equisetum fluviatile* | | *Equisetum hyemale* | 0.00250983 | 0.00236146 | 1.0628298 |
| *matK* | *Equisetum fluviatile* | | *Equisetum ramosissimum* | 0.00178398 | 0.00141456 | 1.2611554 |
| *matK* | *Equisetum fluviatile* | | *E. ramosissimum. debile* | 0.0139409 | 0.01210205 | 1.1519453 |
| *matK* | *Equisetum fluviatile* | | *Equisetum variegatum* | 0.01475409 | 0.01310205 | 1.1260902 |
| *matK* | *Equisetum fluviatile* | | *Equisetum xylochaetum* | 0.01124409 | 0.00910205 | 1.235336 |
| *matK* | *Equisetum fluviatile* | | *Psilotum nudum* | 0.0175409 | 0.01670205 | 1.0502244 |
| *matK* | *Equisetum fluviatile* | | *Ophioglossum vulgatum* | 0.01409 | 0.010205 | 1.3806957 |
| *matK* | *Equisetum fluviatile* | | *Angiopteris fokiensis* | 0.019409 | 0.01710205 | 1.1348932 |
| *matK* | *Equisetum fluviatile* | | *Christensenia aesculifolia* | 0.0179409 | 0.01210205 | 1.4824678 |
| *matK* | *Equisetum fluviatile* | | *Diplopterygium glaucum* | 0.0159409 | 0.00910205 | 1.7513527 |
| *matK* | *Equisetum fluviatile* | | *Osmunda japonica* | 0.00140531 | 0.00139639 | 1.006385 |
| *rpoB* | *Equisetum arvense* | | *Equisetum fluviatile* | 0.001405 | 0.00125494 | 1.1195754 |
| *rpoB* | *Equisetum arvense* | | *Equisetum diffusum* | 0.00105 | 0.00079549 | 1.3199345 |
| *rpoB* | *Equisetum arvense* | | *Equisetum palustre* | 0.00140587 | 0.00119533 | 1.1761404 |
| *rpoB* | *Equisetum arvense* | | *Equisetum pratense* | 0.00095256 | 0.00079431 | 1.1992295 |
| *rpoB* | *Equisetum arvense* | | *Equisetum sylvaticum* | 0.00141377 | 0.00134087 | 1.0543677 |
| *rpoB* | *Equisetum arvense* | | *Equisetum scirpoides* | 0.00627949 | 0.00533024 | 1.1780877 |
| *rpoB* | *Equisetum arvense* | | *Equisetum hyemale* | 0.00987949 | 0.00783024 | 1.2617097 |
| *rpoB* | *Equisetum arvense* | | *Equisetum ramosissimum* | 0.00320871 | 0.00184022 | 1.7436556 |
| *rpoB* | *Equisetum arvense* | | *E. ramosissimum. debile* | 0.00270871 | 0.00196402 | 1.3791648 |
| *rpoB* | *Equisetum arvense* | | *Equisetum variegatum* | 0.00280871 | 0.00264022 | 1.0638167 |
| *rpoB* | *Equisetum arvense* | | *Equisetum xylochaetum* | 0.00290871 | 0.00274022 | 1.0614878 |
| *rpoB* | *Equisetum arvense* | | *Psilotum nudum* | 0.00250871 | 0.0024022 | 1.0443385 |
| *rpoB* | *Equisetum arvense* | | *Ophioglossum vulgatum* | 0.00320871 | 0.00264022 | 1.2153192 |
| *rpoB* | *Equisetum arvense* | | *Angiopteris fokiensis* | 0.03520871 | 0.03164022 | 1.1127833 |
| *rpoB* | *Equisetum arvense* | | *Christensenia aesculifolia* | 0.03320871 | 0.03164022 | 1.0495727 |
| *rpoB* | *Equisetum arvense* | | *Diplopterygium glaucum* | 0.0020871 | 0.00164022 | 1.2724513 |
| *rpoB* | *Equisetum arvense* | | *Osmunda japonica* | 0.00320871 | 0.00264022 | 1.2153192 |
| *rps18* | *Equisetum diffusum* | | *Equisetum fluviatile* | 0.00320871 | 0.00264022 | 1.2153192 |
| *rps18* | *Equisetum diffusum* | | *Equisetum arvense* | 0.001720871 | 0.00164022 | 1.0491708 |
| *rps18* | *Equisetum diffusum* | | *Equisetum palustre* | 0.0022077 | 0.00164269 | 1.3439541 |
| *rps18* | *Equisetum diffusum* | | *Equisetum pratense* | 0.00122077 | 0.00064269 | 1.8994694 |
| *rps18* | *Equisetum diffusum* | | *Equisetum sylvaticum* | 0.00322077 | 0.0014269 | 2.2571799 |
| *rps18* | *Equisetum diffusum* | | *Equisetum scirpoides* | 0.00332077 | 0.00324269 | 1.0240788 |
| *rps18* | *Equisetum diffusum* | | *Equisetum hyemale* | 0.019408 | 0.0158372 | 1.2254691 |
| *rps18* | *Equisetum diffusum* | | *Equisetum ramosissimum* | 0.0129408 | 0.01058372 | 1.2227081 |
| *rps18* | *Equisetum diffusum* | | *E. ramosissimum. debile* | 0.00356909 | 0.00301911 | 1.1821663 |
| *rps18* | *Equisetum diffusum* | | *Equisetum variegatum* | 0.0036909 | 0.00341911 | 1.0794914 |
| *rps18* | *Equisetum diffusum* | | *Equisetum xylochaetum* | 0.00356909 | 0.00315191 | 1.1323575 |
| *rps18* | *Equisetum diffusum* | | *Psilotum nudum* | 0.003909 | 0.00384911 | 1.0155594 |
| *rps18* | *Equisetum diffusum* | | *Ophioglossum vulgatum* | 0.00356909 | 0.0031911 | 1.1184513 |
| *rps18* | *Equisetum diffusum* | | *Angiopteris fokiensis* | 0.0166528 | 0.01131 | 1.4723961 |
| *rps18* | *Equisetum diffusum* | | *Christensenia aesculifolia* | 0.017528 | 0.01701131 | 1.0303733 |
| *rps18* | *Equisetum diffusum* | | *Diplopterygium glaucum* | 0.0166528 | 0.01541131 | 1.0805571 |
| *rps18* | *Equisetum diffusum* | | *Osmunda japonica* | 0.0184528 | 0.01701131 | 1.0847372 |
| *psaA* | *Equisetum palustre* | | *Equisetum fluviatile* | 0.0166528 | 0.01501131 | 1.1093502 |
| *psaA* | *Equisetum palustre* | | *Equisetum arvense* | 0.0176528 | 0.01701131 | 1.0377096 |
| *psaA* | *Equisetum palustre* | | *Equisetum diffusum* | 0.0167063 | 0.01602351 | 1.0426118 |
| *psaA* | *Equisetum palustre* | | *Equisetum pratense* | 0.00877063 | 0.00702351 | 1.2487531 |
| *psaA* | *Equisetum palustre* | | *Equisetum sylvaticum* | 0.0108508 | 0.0090015 | 1.2054435 |
| *psaA* | *Equisetum palustre* | | *Equisetum scirpoides* | 0.0048508 | 0.00390015 | 1.243747 |
| *psaA* | *Equisetum palustre* | | *Equisetum hyemale* | 0.08508145 | 0.07390015 | 1.1513028 |
| *psaA* | *Equisetum palustre* | | *Equisetum ramosissimum* | 0.0098473 | 0.0090015 | 1.0939621 |
| *psaA* | *Equisetum palustre* | | *E. ramosissimum. debile* | 0.0108508 | 0.009724 | 1.1158782 |
| *psaA* | *Equisetum palustre* | | *Equisetum variegatum* | 0.014675 | 0.0132756 | 1.1054114 |
| *psaA* | *Equisetum palustre* | | *Equisetum xylochaetum* | 0.0185713 | 0.01674598 | 1.1090005 |
| *psaA* | *Equisetum palustre* | | *Psilotum nudum* | 0.0218508 | 0.01890015 | 1.1561178 |
| *psaA* | *Equisetum palustre* | | *Ophioglossum vulgatum* | 0.0208849 | 0.01770107 | 1.1798668 |
| *psaA* | *Equisetum palustre* | | *Angiopteris fokiensis* | 0.00884987 | 0.00701066 | 1.2623448 |
| *psaA* | *Equisetum palustre* | | *Christensenia aesculifolia* | 0.00275252 | 0.00260043 | 1.0584865 |
| *psaA* | *Equisetum palustre* | | *Diplopterygium glaucum* | 0.01875143 | 0.01451674 | 1.2917108 |
| *psaA* | *Equisetum palustre* | | *Osmunda japonica* | 0.014143 | 0.013254 | 1.0670741 |
| *accD* | *Equisetum pratense* | | *Equisetum fluviatile* | 0.014143 | 0.01401674 | 1.0090078 |
| *accD* | *Equisetum pratense* | | *Equisetum arvense* | 0.00457143 | 0.00401674 | 1.1380946 |
| *accD* | *Equisetum pratense* | | *Equisetum diffusum* | 0.00124143 | 0.00090167 | 1.3768058 |
| *accD* | *Equisetum pratense* | | *Equisetum palustre* | 0.01357143 | 0.01021674 | 1.3283523 |
| *accD* | *Equisetum pratense* | | *Equisetum sylvaticum* | 0.0157143 | 0.01401674 | 1.1211095 |
| *accD* | *Equisetum pratense* | | *Equisetum scirpoides* | 0.0135745 | 0.01328746 | 1.021602 |
| *accD* | *Equisetum pratense* | | *Equisetum hyemale* | 0.015769841 | 0.01401674 | 1.1250719 |
| *accD* | *Equisetum pratense* | | *Equisetum ramosissimum* | 0.00040545 | 0.00039808 | 1.0185139 |
| *accD* | *Equisetum pratense* | | *E. ramosissimum. debile* | 0.00140545 | 0.00139808 | 1.0052715 |
| *accD* | *Equisetum pratense* | | *Equisetum variegatum* | 0.000545 | 0.00039808 | 1.3690715 |
| *accD* | *Equisetum pratense* | | *Equisetum xylochaetum* | 0.001340498 | 0.00132766 | 1.0096704 |
| *accD* | *Equisetum pratense* | | *Psilotum nudum* | 0.001988471 | 0.00139766 | 1.4227154 |
| *accD* | *Equisetum pratense* | | *Ophioglossum vulgatum* | 0.001574 | 0.00134745 | 1.1681307 |
| *accD* | *Equisetum pratense* | | *Angiopteris fokiensis* | 0.000140514 | 0.000131 | 1.072626 |
| *accD* | *Equisetum pratense* | | *Christensenia aesculifolia* | 0.00117555 | 0.00098476 | 1.1937389 |
| *accD* | *Equisetum pratense* | | *Diplopterygium glaucum* | 0.00564841 | 0.00539763 | 1.0464609 |
| *accD* | *Equisetum pratense* | | *Osmunda japonica* | 0.00141274 | 0.00099763 | 1.4160947 |
| *ndhF* | *Equisetum sylvaticum* | | *Equisetum fluviatile* | 0.00140514 | 0.00129763 | 1.0828502 |
| *ndhF* | *Equisetum sylvaticum* | | *Equisetum arvense* | 0.0013575 | 0.0011476 | 1.1829035 |
| *ndhF* | *Equisetum sylvaticum* | | *Equisetum diffusum* | 0.00136984 | 0.000975 | 1.4049641 |
| *ndhF* | *Equisetum sylvaticum* | | *Equisetum palustre* | 0.00143575 | 0.00139755 | 1.0273365 |
| *ndhF* | *Equisetum sylvaticum* | | *Equisetum pratense* | 0.001621575 | 0.00160755 | 1.0087267 |
| *ndhF* | *Equisetum sylvaticum* | | *Equisetum scirpoides* | 0.0028167 | 0.00279611 | 1.0073631 |
| *ndhF* | *Equisetum sylvaticum* | | *Equisetum hyemale* | 0.007486464 | 0.00679521 | 1.1017263 |
| *ndhF* | *Equisetum sylvaticum* | | *Equisetum ramosissimum* | 0.00281607 | 0.0027912 | 1.0089101 |
| *ndhF* | *Equisetum sylvaticum* | | *E. ramosissimum. debile* | 0.00227554 | 0.00215745 | 1.054738 |
| *ndhF* | *Equisetum sylvaticum* | | *Equisetum variegatum* | 0.00279548 | 0.00249571 | 1.1201141 |
| *ndhF* | *Equisetum sylvaticum* | | *Equisetum xylochaetum* | 0.00270784 | 0.00262671 | 1.0308881 |
| *ndhF* | *Equisetum sylvaticum* | | *Psilotum nudum* | 0.00222707 | 0.00215854 | 1.0317483 |
| *ndhF* | *Equisetum sylvaticum* | | *Ophioglossum vulgatum* | 0.00072844 | 0.00062671 | 1.1623313 |
| *ndhF* | *Equisetum sylvaticum* | | *Angiopteris fokiensis* | 0.00222707 | 0.0019748 | 1.1277446 |
| *ndhF* | *Equisetum sylvaticum* | | *Christensenia aesculifolia* | 0.00244419 | 0.00241449 | 1.0123026 |
| *ndhF* | *Equisetum sylvaticum* | | *Diplopterygium glaucum* | 0.002707274 | 0.00262671 | 1.0306727 |
| *ndhF* | *Equisetum sylvaticum* | | *Osmunda japonica* | 0.00227845 | 0.0021755 | 1.0473225 |
| *psdH* | *Equisetum scirpoides* | | *Equisetum fluviatile* | 0.00222707 | 0.00162671 | 1.3690673 |
| *psdH* | *Equisetum scirpoides* | | *Equisetum arvense* | 0.000707715 | 0.00062671 | 1.1292613 |
| *psdH* | *Equisetum scirpoides* | | *Equisetum diffusum* | 0.002157461 | 0.00154417 | 1.3971696 |
| *psdH* | *Equisetum scirpoides* | | *Equisetum palustre* | 0.00283101 | 0.00279405 | 1.0132288 |
| *psdH* | *Equisetum scirpoides* | | *Equisetum pratense* | 0.00478331 | 0.00423404 | 1.1297272 |
| *psdH* | *Equisetum scirpoides* | | *Equisetum sylvaticum* | 0.00354 | 0.00312444 | 1.1330019 |
| *psdH* | *Equisetum scirpoides* | | *Equisetum hyemale* | 0.003541 | 0.00298443 | 1.1864912 |
| *psdH* | *Equisetum scirpoides* | | *Equisetum ramosissimum* | 0.00493193 | 0.0043284 | 1.1394349 |
| *psdH* | *Equisetum scirpoides* | | *E. ramosissimum. debile* | 0.004875319 | 0.0043284 | 1.126356 |
| *psdH* | *Equisetum scirpoides* | | *Equisetum variegatum* | 0.00493193 | 0.0043284 | 1.1394349 |
| *psdH* | *Equisetum scirpoides* | | *Equisetum xylochaetum* | 0.00147585 | 0.0013284 | 1.1109982 |
| *psdH* | *Equisetum scirpoides* | | *Psilotum nudum* | 0.0015791 | 0.00132727 | 1.1897353 |
| *psdH* | *Equisetum scirpoides* | | *Ophioglossum vulgatum* | 0.0049446 | 0.00476526 | 1.0376353 |
| *psdH* | *Equisetum scirpoides* | | *Angiopteris fokiensis* | 0.001876149 | 0.00132727 | 1.4135396 |
| *psdH* | *Equisetum scirpoides* | | *Christensenia aesculifolia* | 0.0049446 | 0.0047727 | 1.0360173 |
| *psdH* | *Equisetum scirpoides* | | *Diplopterygium glaucum* | 0.00675034 | 0.00642234 | 1.0510717 |
| *psdH* | *Equisetum scirpoides* | | *Osmunda japonica* | 0.007485034 | 0.00642234 | 1.1654683 |
| *psdD* | *Equisetum hyemale* | | *Equisetum fluviatile* | 0.007245034 | 0.00642234 | 1.1280988 |
| *psdD* | *Equisetum hyemale* | | *Equisetum arvense* | 0.0245034 | 0.02144223 | 1.1427634 |
| *psdD* | *Equisetum hyemale* | | *Equisetum diffusum* | 0.0250247 | 0.02464223 | 1.0155208 |
| *psdD* | *Equisetum hyemale* | | *Equisetum palustre* | 0.007825494 | 0.00642234 | 1.2184802 |
| *psdD* | *Equisetum hyemale* | | *Equisetum pratense* | 0.0245034 | 0.0230174 | 1.0645599 |
| *psdD* | *Equisetum hyemale* | | *Equisetum sylvaticum* | 0.008465721 | 0.00642234 | 1.3181677 |
| *psdD* | *Equisetum hyemale* | | *Equisetum scirpoides* | 0.00642234 | 0.01713487 | 0.3748111 |
| *psdD* | *Equisetum hyemale* | | *Equisetum ramosissimum* | 0.0179254 | 0.020314 | 0.8824161 |
| *psdD* | *Equisetum hyemale* | | *E. ramosissimum. debile* | 0.032974846 | 0.07174175 | 0.4596326 |
| *psdD* | *Equisetum hyemale* | | *Equisetum variegatum* | 0.04176151 | 0.09418713 | 0.4433887 |
| *psdD* | *Equisetum hyemale* | | *Equisetum xylochaetum* | 0.0642234 | 0.1171245 | 0.5483345 |
| *psdD* | *Equisetum hyemale* | | *Psilotum nudum* | 0.05023552 | 0.15034415 | 0.3341368 |
| *psdD* | *Equisetum hyemale* | | *Ophioglossum vulgatum* | 0.005054647 | 0.05878154 | 0.0859904 |
| *psdD* | *Equisetum hyemale* | | *Angiopteris fokiensis* | 0.01974654 | 0.09845034 | 0.2005736 |
| *psdD* | *Equisetum hyemale* | | *Christensenia aesculifolia* | 0.00642234 | 0.07545451 | 0.0851154 |
| *psdD* | *Equisetum hyemale* | | *Diplopterygium glaucum* | 0.004187985 | 0.09879454 | 0.0423909 |
| *psdD* | *Equisetum hyemale* | | *Osmunda japonica* | 0.005184165 | 0.09787454 | 0.0529675 |
| *rbcL* | *Equisetum ramosissimum* | | *Equisetum fluviatile* | 0.0245034 | 0.01542234 | 1.588825 |
| *rbcL* | *Equisetum ramosissimum* | | *Equisetum arvense* | 0.00320978 | 0.00311959 | 1.0289099 |
| *rbcL* | *Equisetum ramosissimum* | | *Equisetum diffusum* | 0.00643644 | 0.00623988 | 1.0315006 |
| *rbcL* | *Equisetum ramosissimum* | | *Equisetum palustre* | 0.0024549 | 0.00163988 | 1.4969998 |
| *rbcL* | *Equisetum ramosissimum* | | *Equisetum pratense* | 0.0023451 | 0.00221545 | 1.0585208 |
| *rbcL* | *Equisetum ramosissimum* | | *Equisetum sylvaticum* | 0.00643644 | 0.0063988 | 1.0058824 |
| *rbcL* | *Equisetum ramosissimum* | | *Equisetum scirpoides* | 0.0049195 | 0.00369641 | 1.3308859 |
| *rbcL* | *Equisetum ramosissimum* | | *Equisetum hyemale* | 0.004132456 | 0.00369641 | 1.1179647 |
| *rbcL* | *Equisetum ramosissimum* | | *E. ramosissimum. debile* | 0.0149195 | 0.01315454 | 1.1341712 |
| *rbcL* | *Equisetum ramosissimum* | | *Equisetum variegatum* | 0.078465456 | 0.07516546 | 1.0439031 |
| *rbcL* | *Equisetum ramosissimum* | | *Equisetum xylochaetum* | 0.015458465 | 0.0146514 | 1.0550845 |
| *rbcL* | *Equisetum ramosissimum* | | *Psilotum nudum* | 0.078458464 | 0.075461 | 1.039722 |
| *rbcL* | *Equisetum ramosissimum* | | *Ophioglossum vulgatum* | 0.094871 | 0.09348451 | 1.0148312 |
| *rbcL* | *Equisetum ramosissimum* | | *Angiopteris fokiensis* | 0.0149195 | 0.01348741 | 1.1061798 |
| *rbcL* | *Equisetum ramosissimum* | | *Christensenia aesculifolia* | 0.09794154 | 0.08157451 | 1.200639 |
| *rbcL* | *Equisetum ramosissimum* | | *Diplopterygium glaucum* | 0.05348465 | 0.0369641 | 1.446935 |
| *rbcL* | *Equisetum ramosissimum* | | *Osmunda japonica* | 0.002454154 | 0.00241548 | 1.0160117 |
| *ycf1* | *E. ramosissimum. debile* | | *Equisetum fluviatile* | 0.079844165 | 0.0669641 | 1.1923428 |
| *ycf1* | *E. ramosissimum. debile* | | *Equisetum arvense* | 0.0149195 | 0.01369641 | 1.0893 |
| *ycf1* | *E. ramosissimum. debile* | | *Equisetum diffusum* | 0.017845465 | 0.0169641 | 1.0519547 |
| *ycf1* | *E. ramosissimum. debile* | | *Equisetum palustre* | 0.03454512 | 0.03369641 | 1.025187 |
| *ycf1* | *E. ramosissimum. debile* | | *Equisetum pratense* | 0.048164 | 0.04469641 | 1.077581 |
| *ycf1* | *E. ramosissimum. debile* | | *Equisetum sylvaticum* | 0.0149195 | 0.0134545 | 1.1088855 |
| *ycf1* | *E. ramosissimum. debile* | | *Equisetum scirpoides* | 0.0187941 | 0.0176464 | 1.0650388 |
| *ycf1* | *E. ramosissimum. debile* | | *Equisetum hyemale* | 0.048794161 | 0.03494641 | 1.3962568 |
| *ycf1* | *E. ramosissimum. debile* | | *Equisetum ramosissimum* | 0.0979464 | 0.09614541 | 1.0187319 |
| *ycf1* | *E. ramosissimum. debile* | | *Equisetum variegatum* | 0.00674614 | 0.00661456 | 1.0198918 |
| *ycf1* | *E. ramosissimum. debile* | | *Equisetum xylochaetum* | 0.0283889 | 0.02484646 | 1.1425732 |
| *ycf1* | *E. ramosissimum. debile* | | *Psilotum nudum* | 0.023465464 | 0.02215464 | 1.059167 |
| *ycf1* | *E. ramosissimum. debile* | | *Ophioglossum vulgatum* | 0.0678946 | 0.0668289 | 1.0159467 |
| *ycf1* | *E. ramosissimum. debile* | | *Angiopteris fokiensis* | 0.0283889 | 0.02714967 | 1.0456442 |
| *ycf1* | *E. ramosissimum. debile* | | *Christensenia aesculifolia* | 0.0987946 | 0.0846464 | 1.1671447 |
| *ycf1* | *E. ramosissimum. debile* | | *Diplopterygium glaucum* | 0.0083889 | 0.0068289 | 1.2284409 |
| *ycf1* | *E. ramosissimum. debile* | | *Osmunda japonica* | 0.097946546 | 0.068289 | 1.4342946 |
| *ycf2* | *Equisetum variegatum* | | *Equisetum fluviatile* | 0.0283889 | 0.016796 | 1.6902179 |
| *ycf2* | *Equisetum variegatum* | | *Equisetum arvense* | 0.007845441 | 0.0068289 | 1.1488587 |
| *ycf2* | *Equisetum variegatum* | | *Equisetum diffusum* | 0.00978965 | 0.0068289 | 1.4335617 |
| *ycf2* | *Equisetum variegatum* | | *Equisetum palustre* | 0.0283889 | 0.027464 | 1.0336768 |
| *ycf2* | *Equisetum variegatum* | | *Equisetum pratense* | 0.007464 | 0.0068289 | 1.0930018 |
| *ycf2* | *Equisetum variegatum* | | *Equisetum sylvaticum* | 0.0283889 | 0.024654 | 1.1514927 |
| *ycf2* | *Equisetum variegatum* | | *Equisetum scirpoides* | 0.067465 | 0.06145874 | 1.0977283 |
| *ycf2* | *Equisetum variegatum* | | *Equisetum hyemale* | 0.0087545 | 0.0068289 | 1.2819781 |
| *ycf2* | *Equisetum variegatum* | | *Equisetum ramosissimum* | 0.0283889 | 0.0279464 | 1.0158339 |
| *ycf2* | *Equisetum variegatum* | | *E. ramosissimum. debile* | 0.00794641 | 0.0068289 | 1.1636442 |
| *ycf2* | *Equisetum variegatum* | | *Equisetum xylochaetum* | 0.0283889 | 0.02165464 | 1.3109846 |
| *ycf2* | *Equisetum variegatum* | | *Psilotum nudum* | 0.046464 | 0.0410165 | 1.1328124 |
| *ycf2* | *Equisetum variegatum* | | *Ophioglossum vulgatum* | 0.06764164 | 0.05987979 | 1.1296238 |
| *ycf2* | *Equisetum variegatum* | | *Angiopteris fokiensis* | 0.0346464 | 0.03389783 | 1.0220831 |
| *ycf2* | *Equisetum variegatum* | | *Christensenia aesculifolia* | 0.0163663 | 0.01474648 | 1.1098447 |
| *ycf2* | *Equisetum variegatum* | | *Diplopterygium glaucum* | 0.087641655 | 0.08416346 | 1.0413267 |
| *ycf2* | *Equisetum variegatum* | | *Osmunda japonica* | 0.00796469 | 0.00789783 | 1.0084656 |
